# Enhancing GABAergic signaling ameliorates aberrant gamma oscillations of olfactory bulb in AD mouse models

**DOI:** 10.1186/s13024-021-00434-7

**Published:** 2021-03-04

**Authors:** Ming Chen, Yunan Chen, Qingwei Huo, Lei Wang, Shuyi Tan, Afzal Misrani, Jinxiang Jiang, Jian Chen, Shiyuan Chen, Jiawei Zhang, Sidra Tabassum, Jichen Wang, Xi Chen, Cheng Long, Li Yang

**Affiliations:** 1 Precise Genome Engineering Center, School of Life Sciences, Guangzhou University, Guangzhou, 510006 China; 2 Department of Pharmacology, Key Laboratory of Anti-inflammatory and Immunopharmacology, School of Basic Medical Sciences, Anhui Medical University, Hefei, 230032 China; 3 School of Life Sciences, South China Normal University, Guangzhou, 510631 China; 4 Institute for Brain Research and Rehabilitation, South China Normal University, Guangzhou, 510631 China; 5 Medical College of Acu-Moxi and Rehabilitation, Guangzhou University of Chinese Medicine, Guangzhou, China; 6 School of Psychology and Center for Studies of Psychological Application, South China Normal University, Guangzhou, 510631 China

**Keywords:** Alzheimer’s disease, APP/PS1, 3xTg, Olfactory bulb, Gamma oscillations, GABA_A_R, Tiagabine

## Abstract

**Background:**

Before the deposition of amyloid-beta plaques and the onset of learning memory deficits, patients with Alzheimer’s disease (AD) experience olfactory dysfunction, typified by a reduced ability to detect, discriminate, and identify odors. Rodent models of AD, such as the Tg2576 and APP/PS1 mice, also display impaired olfaction, accompanied by aberrant in vivo or in vitro gamma rhythms in the olfactory pathway. However, the mechanistic relationships between the electrophysiological, biochemical and behavioral phenomena remain unclear.

**Methods:**

To address the above issues in AD models, we conducted in vivo measurement of local field potential (LFP) with a combination of in vitro electro-olfactogram (EOG), whole-cell patch and field recordings to evaluate oscillatory and synaptic function and pharmacological regulation in the olfactory pathway, particularly in the olfactory bulb (OB). Levels of protein involved in excitation and inhibition of the OB were investigated by western blotting and fluorescence staining, while behavioral studies assessed olfaction and memory function.

**Results:**

LFP measurements demonstrated an increase in gamma oscillations in the OB accompanied by altered olfactory behavior in both APP/PS1 and 3xTg mice at 3–5 months old, i.e. an age before the onset of plaque formation. Fewer olfactory sensory neurons (OSNs) and a reduced EOG contributed to a decrease in the excitatory responses of M/T cells, suggesting a decreased ability of M/T cells to trigger interneuron GABA release indicated by altered paired-pulse ratio (PPR), a presynaptic parameter. Postsynaptically, there was a compensatory increase in levels of GABA_A_R α1 and β3 subunits and subsequent higher amplitude of inhibitory responses. Strikingly, the GABA uptake inhibitor tiagabine (TGB) ameliorated abnormal gamma oscillations and levels of GABA_A_R subunits, suggesting a potential therapeutic strategy for early AD symptoms. These findings reveal increased gamma oscillations in the OB as a core indicator prior to onset of AD and uncover mechanisms underlying aberrant gamma activity in the OB.

**Conclusions:**

This study suggests that the concomitant dysfunction of both olfactory behavior and gamma oscillations have important implications for early AD diagnosis: in particular, awareness of aberrant GABAergic signaling mechanisms might both aid diagnosis and suggest therapeutic strategies for olfactory damage in AD.

**Supplementary Information:**

The online version contains supplementary material available at 10.1186/s13024-021-00434-7.

## Background

Alzheimer’s disease (AD) is the most prevalent form of dementia in the elderly [1, 2]. The hallmarks of AD include senile plaques, composed primarily of amyloid-beta (Aβ) protein, as well as neurofibrillary tangles and memory loss [3–5]. Clinical trials of potential therapies for AD have thus far met with very limited success [3, 6, 7]. Therefore, there is still much research interest in establishing methods to diagnose and prevent AD before the onset of the irreversible deterioration phase of the disease. Although the primary sensory centers of the brain are minimally affected [8], patients with early-stage AD exhibit olfactory perceptual deficits, often coinciding with, or preceding, the manifestation of classical cognitive impairments such as memory loss [9–13]. Thus, one potential approach to the early diagnosis of AD would be to detect the olfactory sensory dysfunction in combination with neuropsychological measures involving affective changes [14, 15].

In the olfactory system, odor is first received by olfactory sensory neurons (OSNs) located in the olfactory epithelium (OE) [16, 17]. After the OSNs convert the chemical signal of the odorant into electrical potential, odor information is transferred to the olfactory bulb (OB) where it is encoded by OB output neurons, mitral/tufted (M/T) cells, and then sent to highly plastic olfactory cortical areas, including the piriform cortex (PC) [18, 19]. AD pathogenic factors, including Aβ aggregation, have been found within the OE, OB and PC in both AD patients and AD rodent models [20–23]. It is now evident that patients with early-stage AD often have a reduced ability to detect, discriminate, and identify odors, coupled with abnormal odor coding [9, 24, 25]. However, potential olfactory biomarkers and the precise neural mechanisms underlying the olfactory deficits in early AD remain poorly understood. Therefore, the usefulness of olfactory screens as an approach to AD diagnosis is hampered by a lack of knowledge on how and when AD pathogenesis impacts olfaction.

Gamma oscillations (40–100 Hz), resulting from activation of excitatory and fast-spiking inhibitory local circuits, have been shown to be necessary for higher cognitive functions and sensory procession [26–28]. Gamma rhythms recruit both neuronal and glial responses to attenuate AD-associated pathology [26, 29] and improve cognition [30], suggesting they could play an important role in AD pathogenesis and treatment. As the first relay of the olfactory system, proper gamma oscillations in the OB are required for odor discrimination and odor learning [27, 31]. Though aberrant gamma rhythms are known to occur in the OB of a Swedish mutation AD model, Tg2576 mice, and OB slices of APP/PS1 mice at ages before Aβ deposition [23, 32], the mechanism and relationship between altered gamma oscillations and local- or long-range-circuitry pathology remain unclear.

In the present study, impaired olfactory detection occurred in 3–5 month-old AD mouse models, including APP/PS1 and 3xTg mice, accompanied by increased gamma oscillations, which may be attributed to a disturbance in the excitation/inhibition (E/I) ratio of OB. Moreover, we discovered that abnormal number of OSNs and subsequent OE → OB excitation, altered glutamatergic- and GABAergic-synaptic transmission and levels of GABA_A_Rs underlie aberrant gamma oscillations. Furthermore, an increase in levels of GABA in the synaptic cleft by blockade of GABA-uptake transporter 1 (GAT1) with tiagabine (TGB), an anti-convulsive medication, attenuated aberrant gamma oscillations in both APP/PS1 and 3xTg mice. The results highlight the potential for the early diagnosis of AD by identification of altered olfactory perception with aberrant gamma oscillatory activity and levels of GABA receptors, and the use of an anti-convulsant medicine, TGB, in the treatment of certain symptoms of early AD. Evidence reviewed here in the context of the emergence of other typical pathological changes in AD suggests that olfactory impairments could be probed to understand the molecular mechanisms involved in the early phases of the pathology.

## Materials and methods

### Animals

All experimental procedures were approved by the Guangzhou University Animal Care and Use Committee. Amyloid precursor protein/presenilin 1 (APP/PS1) double transgenic mice derived from the B6C3-Tg (APPswe, PSEN1dE9) 85Dbo/J (JAX 004462), which expresses a chimeric mouse/human APP gene (APPswe) and human mutant PS1 (DeltaE9), were obtained from the Model Animal Research Center of Nanjing University. 3xTg mice (MMRRC stock #34830) expressing three mutations associated with familial Alzheimer’s disease (APP Swedish, MAPT P301L, and PSEN1 M146V) [33] are a gift from Dr. Lingqiang Zhu at Huazhong University of Science and Technology. The mice were maintained at South China Normal University according to SPF standards and were genotyped by polymerase chain reaction (PCR) according to the Jackson Laboratory (JAX) protocol. Animals were housed in cages in which mice could eat and drink freely, with a 12-h light/dark cycle. All animals involved in experiments were 3–5 month-old unless otherwise indicated, and were used according to international and our university’s ethical standards.

### Behavioral test

#### Cookie-finding test

An eight-arm radial maze was used to conduct a cookie-finding test to evaluate olfactory function [34]. Both male and female mice, naive to the test prior to the first trial, were used. Briefly, mice were trained to explore freely in the maze for 5 min/day for five consecutive days. Mice were next deprived of food overnight with sufficient access to water. In the morning of day six, mouse was placed in the maze, and one piece of cookie (3 g; Nutter Butter, Nabisco) was placed at the distal end of one arm of the maze with a blocker. Each mouse was given one test per day. The cookie-finding test was conducted for five consecutive days and the food arm was changed every day so the animal could not rely on spatial memories to find the cookie. The times of the animal entering each arm were calculated, and the averaged numbers of wrong arms entered/day were used to evaluate olfactory ability. Each trial was recorded by a video tracking system and analyzed using the manufacturer’s software (Zhenghua Instruments).

#### Buried food test

A buried food test was performed as described previously [32, 35]. Briefly, the three-days protocol consists of an odor familiarization exercise on day 1, food deprivation on day 2 and testing on day 3. Mice were individually introduced into a clean cage containing 3 cm deep of clean bedding with a small piece (10 mm cube) of peanut chocolate buried beneath 1 cm in a random corner of the cage. Time spent to retrieve the cookie (latency) was measured. If the mouse failed to find the buried food within 5 min, the test was stopped, and the latency score was recorded as 300 s.

#### Working memory test

Delayed spatial win-shift (SWSh) paradigm was used to assess working memory function in rodents [36, 37]. On the first 2 days of testing, mice were placed in the maze and allowed to explore for 5 min with no food available, after which they were returned to their home cages with a little food. Subsequent SWSh trials were given once daily. These trials consisted of a training phase and a test phase, separated by a 5-min delay. Within-phase error that the mice re-enter each arm was considered as a working memory error, while failure to only visit the arms containing food in the test phase was defined as across-phase (reference memory) error. All mice were allowed a maximum of 5 min to retrieve the four pellets during the test phase. The maze was cleaned with 15% ethanol after each test.

#### Open field test (OFT)

The OFT was performed in the same way as described [38, 39]. Briefly, the apparatus consisted of a gray square subdivided into four 50 cm × 50 cm × 40 cm zones. Each zone was divided into 3 × 3 equal squares, which had been drawn on the floor of the arena. The test room was dimly illuminated. After 30 s adaptation, a single mouse was subjected to the OFT for 10 min and its behavior was recorded using a video camera located 120 cm above the arena under normal lighting conditions (800 lx).

### Electrophysiology

#### Electro-olfactogram (EOG) recording

EOGs were recorded in the OE located on the septum of adult mice according to previously described methods [34, 40]. The animal was sacrificed and sagittally hemisected to expose the septum, which is covered with OE. The halved head was placed on a mounting dish in which the reference electrode was embedded. Additionally, the Ag/AgCl recording wire was put into glass electrode filled with Ringer solution (140 mM NaCl, 5 mM KCl, 2 mM CaCl_2_, 1 mM MgCl_2_, 10 mM HEPES and 10 mM glucose; pH 7.2) placed directly on the epithelium surface. A picospritzer (Parker Instrumentation, USA) was used to eject amyl acetate (1 mM dilution in Ringer for 100 ms) filled in glass micropipette directly onto the OE. Data were filtered during acquisition with a low pass filter set at 2 kHz using pClamp10 (Molecular Devices) and were analyzed offline with Clampfit 10.2 (Molecular Devices).

#### In vivo extracellular recording

In vivo surgery/extracellular recordings were conducted as described [41]. In brief, mice were anesthetized by intraperitoneal (IP) injection with urethane (2 g/kg; Sigma-Aldrich). Anesthetic depths in mice were monitored by checking the tail/toe-pinch reflex and respiratory rate. The anesthetized animal was next positioned in a stereotaxic apparatus (RWD Life Science) for surgery, and LFP recordings were usually conducted between 0.5–1 h after anesthesia, with body temperature maintained at 37 ± 0.5 °C by a heating pad with feedback temperature control (Harvard Apparatus). A small skin incision was made in the scalp and the skull was exposed under a stereomicroscope (Zeiss). The glass microelectrodes used for recording (1–2 MΩ resistances for OB and 3–5 MΩ resistances for anterior piriform cortex, aPC) were pulled from borosilicate glass capillary tubes with an outer diameter of 1.0 mm (Nanjing) and filled with 500 mM NaCl. After the dura mater was removed, the microelectrodes were advanced and further lowered with mechanical micromanipulators (Narishige) to dual-site coordinates: AP: 3.9 mm; ML: 0.3 mm; vertical: 0.8–0.9 mm for OB and AP: 1.8 mm; ML: 2.5 mm; vertical: 3.5–3.8 mm for aPC. The stereotaxic coordinates were carefully manipulated to make the recording sites consistent among animals and histological confirmation of recording sites were done after experiments. Possible variations of recording sites and timing did not show significant differences in OB gamma oscillations (Fig. S1). Single-site recording in dorsal hippocampus was made as below: 1.8 mm posterior to the bregma, 1.25 mm lateral to the midline, and to a depth of 1.1 mm. Acquisition of LFP was started after the electrical signals were stable with depth of anesthesia maintaining as loss of a toe-pinch reflex. Each recorded signal was amplified (1000 x) and band-pass filtered (0.1–1000 Hz) by an electrometer amplifier (Model 3000; A-M Systems), then digitized via a D/A converter (Micro 1401; Cambridge Electronic Design) and Spike2 software (Cambridge Electronic Design).

#### Whole-cell patch clamping and field recordings in vitro

Whole-cell responses were recorded as described previously [42, 43]. Briefly, coronal or horizontal slices (320–350 μm thick) of OB were cut using a vibratome (VT1000S; Leica) for patch clamp and field recording, respectively. Ice-cold cutting solution containing the following: 93 mM N-methyl-D-glucamine, 93 mM HCl, 2.5 mM KCl, 1.2 mM NaH_2_PO_4_, 30 mM NaHCO_3_, 20 mM HEPES, 25 mM glucose, 5 mM (+)-sodium L-ascorbate, 2 mM thiourea, 3 mM sodium pyruvate, 0.5 mM CaCl_2_, 10 mM MgSO_4_ (pH 7.4). Slices were first incubated for 10 min in cutting solution at 32 ± 0.5 °C, then transferred to incubating solution containing the following: 92 mM NaCl, 2.5 mM KCl, 1.2 mM NaH_2_PO_4_, 30 mM NaHCO_3_, 20 mM HEPES, 25 mM glucose, 5 mM (+)-sodium L-ascorbate, 2 mM thiourea, 3 mM sodium pyruvate, 2 mM CaCl_2_, 2 mM MgSO_4_ for 1 h at room temperature (RT). The artificial cerebral spinal fluid (aCSF) for whole-cell and field recording contains the following: 124 mM NaCl, 2.5 mM KCl, 2 mM CaCl_2_, 1.2 mM NaH_2_PO_4_, 24 mM NaHCO_3_, 2 mM MgSO_4_, 12.5 mM D-glucose, 5 mM HEPES (pH 7.3). Both incubating and recording solutions were oxygenated with 95% O_2_/5% CO_2_ throughout the experiment. Pipettes (3–5 MΩ resistances) were filled with internal solution consisting of 100 mM cesium methane sulfonate, 10 mM NaCl, 10 mM tetraethylammonium chloride (TEA-Cl), 10 mM HEPES, 1 mM MgCl_2_, 2 mM Mg-ATP, 0.3 mM Na-GTP, 4 mM QX-314 (pH 7.3). For miniature excitatory postsynaptic current (mEPSC) and miniature inhibitory postsynaptic current (mIPSC) recordings, the aCSF was supplemented with 1 μM tetrodotoxin (TTX). Single and paired-pulse responses were induced by local electrical stimulation using a concentric bipolar electrode (WPI, Inc) for patch-clamping (in the presence of 50 μM APV and 20 μM CNQX) or field recording, respectively. Picrotoxin (Sigma, P1675) at 100 μM or baclofen (Sigma, B5399) at 50 μM was used whenever necessary. Data were cut off with a low-pass filter (2 kHz) using an amplifier (Axopatch 700B; Axon Instruments), then digitized by pClamp10 (Molecular Devices). Data analysis was performed offline with Mini Analysis (Synaptosft) or Clampfit10 software (Molecular Devices).

#### Brain homogenate puff assay

We performed brain homogenate puff assays as we reported [44]. Briefly, animals were euthanized by fast cervical dislocation, and the brains were quickly removed, then washed in ice–cold phosphate-buffered saline (PBS). OBs of WT or APP/PS1 mice were dissected, weighed, and flash-frozen in liquid nitrogen and then stored at − 80 °C. To make OB homogenate, two freeze–thaw cycles were performed to break the cell membranes before adding 20 μl bath solution per mg tissue into the tube, followed by sonication for 1 min on ice. The homogenate was centrifuged for 5 min at 5000 rpm, and the supernatant of WT or APP/PS1 was then aliquoted and stored at − 20 °C. The stored OB supernatant was further diluted using aCSF (1:100) for puffing experiments. Supernatant-puff on OB slices were of 200 ms duration at 50 psi and were delivered using a Picospritzer (Parker Instrumentation, USA). Puff-evoked whole cell IPSCs were evaluated on OB slices of WT mice. Although the OB supernatant contains both glutamate and GABA, the experiments were performed in the presence of 50 μM APv and 20 μM CNQX to block the excitatory response, and thus the puff-induced responses represent the GABA-induced response only.

### Immunofluorescence staining

For OE staining, animals were decapitated followed by skin stripping and removal of brain tissue. The turbinate was separated and immersed in 0.5 M EDTA (pH 8.0) solution at RT for 2 weeks. Paraffin-embedded OE tissue was sectioned into 4 μm thick slices, then mounted on gelatin-coated slides. The slides were incubated for 12 h at 4 °C with olfactory marker protein (OMP) primary antibody (Abcam, ab62609, 1:200; Santa Cruz, sc365818, 1:100) in a humidified chamber, then washed and incubated for 50 min with goat anti-rabbit secondary antibody (KPL, 074–1505) or goat anti-mouse secondary antibody (Servicebio, GB21301). Next, sections were washed and incubated with 4, 6-diamidino-2-phenylindole dihydrochloride (DAPI; Vectashield) for 15 min. Images were captured with a light microscope using 20X and 40X magnification. For other protein staining, mice were perfused transcardially with 0.9% saline and 4% paraformaldehyde (PFA) in PBS (pH 7.4). After perfusion, brains were post-fixed in 4% PFA for 3 h, dehydrated in 30% sucrose overnight and then sectioned at 30 μm (for c-Fos staining), 20 μm (for PV, GFAP and Aβ1–42 staining) and 10 μm (for GABA_A_R α1, β3 and α5 staining), respectively, using a freezing microtome (Leica). Sections were washed three times with PBS, blocked with 0.3% Triton X-100 (Sigma-Aldrich) containing 5% normal bovine serum (Roche, 38,680,326) and then incubated with primary antibodies (Table 1) which were diluted in PBS + 10% TritonX-100 + 3% BSA. After incubation with primary antibody for 24 h at 4 °C, sections were washed in PBS three times for 10 min each and incubated with goat-anti-rabbit secondary antibody (KPL, 5570–0007) diluted in PBS + 10% Triton X-100 + 3% BSA for 1 h at RT. Sections were subsequently washed and mounted on glass slides, then imaged on a fluorescence microscope (EVOS) or a laser confocal microscope (Nikon). Images were analyzed using Image J software (National Institutes of Health). For each section, cell counting was performed whereby the cell number was normalized to the neuronal layer area.

**Table 1:** Information of the primary antibodies used in this study

Antigen	Source	Catalog number	Host species	Dilution	
				IF	WB
Aβ 1–42	Abcam	ab201060	Rabbit	1:200	
c-Fos	Abcam	ab190289	Rabbit	1:1000	
GABAAR-α1	Millipore	06–868	Rabbit	1:1000	1:5000
GABAAR-α5	Abcam	ab175195	Rabbit	1:500	1:5000
GABAAR-β2	Abcam	ab156000	Rabbit		1:10000
GABAAR-β3	Novus Biologicals	NB300–199	Rabbit	1:300	1:5000
GABA_B1_R	Abcam	ab55051	Rabbit		1: 5000
GABA_B1_R	Affinity	AF0162	Rabbit		1:2000
GABA_B2_R	Neuromab	75–124	Rabbit		1:2500
GAD65/67	Abcam	ab11070	Rabbit		1:5000
GADPH	Bioworld	MB001H	Mouse		1:5000
GAT1	Abcam	ab426	Rabbit		1:500
GAT3	Abcam	ab431	Rabbit		1:1000
GFAP	Thermo Fisher	13–0300	Mouse	1:1000	
GluR1	Abcam	ab31232	Rabbit		1:5000
GluR2	Abcam	ab206293	Rabbit		1:2000
mGluR5	Millipore	MABN540	Mouse		1:2000
OMP	Abcam	ab62609	Rabbit	1:200	
OMP	Santa Cruz	sc365818	Mouse	1:100	
PV	Abcam	ab11427	Rabbit	1:1000	
VGAT	Abcam	ab101999	Mouse		1:1000
NMDAR1	Abcam	ab109182	Rabbit		1:5000
β-CTF	Sigma	A8717	Rabbit		1:4000
γ-tubulin	Abcam	T6557	Mouse		1:10000

### Western blotting

Mouse brains were dissected on ice and the OB tissues were homogenized in lysis buffer containing 50 mM Tris, pH 7.5, 150 mM NaCl, 1% SDS, 5 mM EDTA and protease inhibitors (Complete Mini; Roche). After centrifugation at 14000 rpm for 20 min at 4 °C, the supernatant was collected for western blotting. Sodium dodecyl sulphate-polyacrylamide gel electrophoresis (SDS-PAGE) was performed using a 5% stacking gel and a 10% separation gel, ran at 80 V for 0.5 h and at 100 V for 1.5 h. Proteins were then electro-transferred to nitrocellulose membranes in transfer buffer at 180 mA for 1.5 h. Membranes were blocked with 5% defatted milk in Tris-buffered saline with Tween 20 (TBST) for 30 min and incubated overnight with primary antibodies, and anti-γ-tubulin antibody (Sigma, T6557) or GADPH (Bioworld, MB001H) was used as loading control (Table 1). After three washes with TBST, incubation with secondary antibody (CWS, cw0104) at RT for 1 h using 5% milk in TBST followed by three additional washes with TBST. Protein bands were visualized using Immobilon Western ECL system (Bio-Rad) and analyzed with Gel-Pro Analysis software (Media Cybernetics).

### Enzyme-linked immunosorbent assay (ELISA)

OBs were dissected and homogenized in 20 μl ice–cold PBS per mg tissue followed by centrifugation for 5 min at 5000 g. Supernatant was aliquoted and stored at − 20 °C. ELISA kit (MEIMIAN, MM-0442 M2) was used for the quantitative determination of GABA. GABA standards and samples were added to the wells of assay plates and incubated for 1 h at 37 °C. Blank wells were added with standard diluent. The horseradish peroxidase (HRP) conjugated reagent (100 μl) was added to each well for 1 h at 37 °C. Plates were washed four times with PBS followed by incubation in chromogen solution (100 μl) for 15 min at 37 °C in the dark. The absorbance at 450 nm, in stop solution (50 μl), was examined with a microplate reader (SPECTRAMAX 190, USA).

### Microinjection and intraperitoneal injection

Chemicals for injection were prepared in advance and stored at − 4 °C or − 20 °C. The GABA_A_R antagonist, GABAzine (GBZ, Sigma-Aldrich, 95,531), was dissolved in saline at 10, 20 or 500 μM for use [27]. TGB (Meilun) was dissolved in absolute ethanol at 266 mM and then diluted with saline to 80 mM for use [43]. After recording extracellular baseline activities for 10 min, GBZ (4 μl) or TGB (2 μl) were locally infused into the OB at a rate of 0.5 μl/min, using a syringe infusion microinjector (Stoelting) with a glass pipette (tip diameter: 5–10 μm). For chronic treatments, TGB or vehicle were IP administrated at a dose of 5 mg/kg once daily for 1 week. The γ-secretase inhibitor, LY-411575 (Sigma, SML0506) was dissolved in DMSO at 100 mg/ml, then emulsified in sunflower oil at 1 mg/ml as described [45]. Mice were IP injected once daily for two consecutive days with LY-411575 (3 mg/kg) or vehicle [45, 46].

### LFP analysis

LFP data were imported and analyzed offline using custom-written tools in MATLAB (MathWorks). Data were first low-pass filtered (300 Hz cutoff) using a fourth-order Butterworth filter to extract LFPs with phase preserving.

#### Power spectral density

Power spectral density was calculated using Thomson’s multitaper method. The resulting spectra were calculated under a fast Fourier transformation length of 2^12^ and were used to determine the area power for the theta (1–10 Hz), beta (12–35 Hz), low gamma (40–70 Hz) and high gamma (70–100 Hz) frequency bands.

#### Coherence

The cross-spectral coherence (CSC) between the two LFP signals from the OB and aPC was analyzed in line with our described method [41]. Using the bispectral function, the calculation in frequencies was performed according to the following formula [47]:

$$ {CSC}_{XY}(f)=\frac{G_{XY}{(f)}^2}{G_{XX}(f)\kern0.5em \cdotp \kern0.5em {G}_{YY}(f)} $$

Coherence values ranged from 0 to 1. CSC = 1 indicates completely correlated in the frequency domain, while CSC = 0 indicates a lack of correlation.

#### Phase synchronization clustering (PSC)

To compute how synchronous between the oscillations of OB and aPC in phase ranges [48], both OB and aPC signals were filtered into different identical bands of interest, then PSC analysis was applied as follows:

$$ PSC=\frac{\sum_{n=1}^N{e}^{i\left[\triangle \upphi (n)\right]}}{N} $$

where △ϕ is the phase difference transformed from the bandpass signal at any point. The *PSC* distributions were determined significance with Rayleigh test for non-uniformity.

#### Phase-amplitude coupling (PAC)

Relation between circular phase and linear amplitude variables across frequencies was evaluated based on linearizing the phase variable into sine and cosine components. First, instantaneous phases were obtained from signals as mentioned above. Subsequently, OB phase was selected from the corresponding transient gamma event of aPC signal. PAC was defined by [49]:

$$ \rho =\sqrt{\frac{{r^2}_{sa}+{r^2}_{ca}-2{r}_{sa}{r}_{ca}{r}_{sc}}{1-{r^2}_{sc}}} $$

where *r*_*sa*_ is the correlation between sin of the phase and amplitude, *r*_*ca*_ is the correlation between cos of the phase and amplitude, and *r*_*sc*_ is the correlation between sin of the phase and cos of the phase. Two oscillations were considered to be significantly coupled if more than 95% of the calculated *ρ* values significant, then the strength was estimated as described [50].

### Statistics

Data were subjected to *t*-test, one-way or two-way analysis of variance (ANOVA) followed by Tukey’s post hoc test (one-way) or Bonferroni post-hoc (two-way) correction, as appropriate with *p* < 0.05 as statistically significant. Nonparametric Kolmogorov-Smirnov test was used for comparing PPR and E/I ratio. For the oscillatory analysis, nonparametric Wilcoxon rank-sum test was performed if the data sets were distributed in a non-Gaussian fashion. All data were expressed as mean ± standard error of the mean (SEM), unless otherwise stated.

## Results

### Impaired olfactory behavior in APP/PS1 and 3xTg mice

**Fig. 1:**
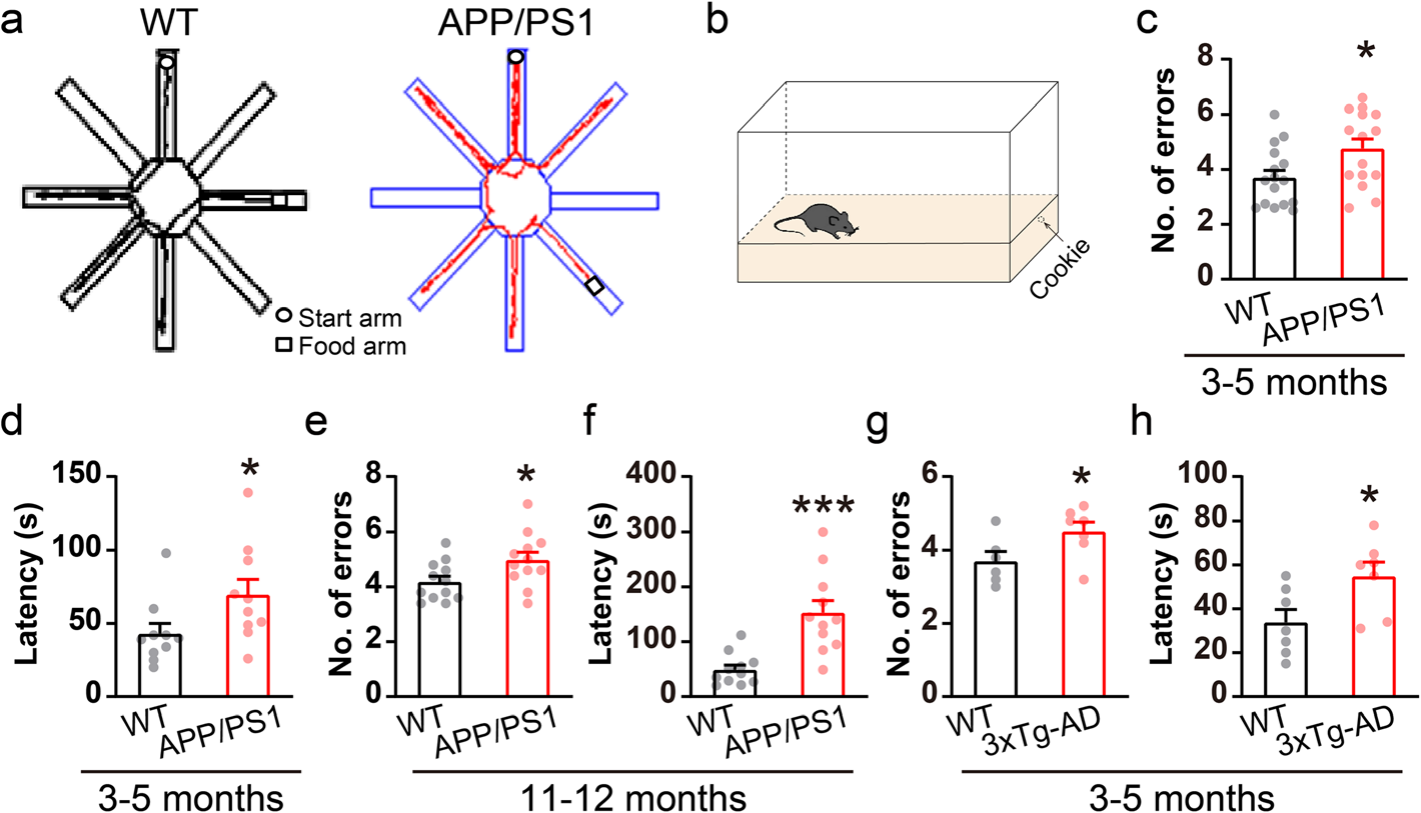
Impaired olfactory behavior in APP/PS1 and 3xTg mice. **a** Representative tracking paths in the cookie-finding test. **b** Illustration of buried-food test. **c** APP/PS1 mice showed significantly more entries into the wrong arms in the cookie-finding test compared to WT littermates (*n* = 16 for WT and 15 for APP/PS1, *p* = 0.02). **d** APP/PS1 mice spent significantly more time to find the food in the buried-food test (*n* = 10 for both WT and APP/PS1, *p* = 0.04). **e** Aged (11–12 months) APP/PS1 mice showed significantly more entries into the wrong arms (*n* = 12 for both WT and APP/PS1, *p* = 0.03). **f** Aged APP/PS1 mice showed significantly increased time spent in finding the food (*n* = 11 for both WT and APP/PS1, *p* = 0.0003). **g**-**h** 3xTg mice showed impaired olfactory behavior as well (*n* = 7 for WT and 3xTg, *p* = 0.04 for cookie-finding test and 0.03 for buried-food test). Values represent mean ± SEM. Two-sample t-test. *p < 0.05, *** *p* < 0.001

Since olfactory deficits occur early in AD pathogenesis [14, 25], we first sought to determine the earliest age at which APP/PS1 mice show impairment in olfaction. Cookie-finding ability was evaluated in young APP/PS1 mice compared to WT controls using a modified eight-arm radial maze (Fig. 1a). In addition to using a blocker to make the cookie invisible, the food arm was changed randomly every day so that mice could not rely on spatial memory. The results showed that the number of entries into incorrect arms was significantly increased in 3–5 month-old APP/PS1 mice (Fig. 1c, WT: 3.7 ± 0.3, APP/PS1: 4.8 ± 0.4), indicating a possible impairment in odor identification in APP/PS1 mice. We next performed a buried-cookie test to confirm these deficits in olfactory function (Fig. 1b), and observed a significantly increased time spent in finding the buried food in APP/PS1 mice compared to WT controls (Fig. 1d, WT: 43 ± 7.02 s, APP/PS1: 69.7 ± 10.4 s). Moreover, olfaction deficits persisted in 11–12 month-old APP/PS1 mice (Fig. 1e-f). While no significant difference was observed in the cookie-finding and buried-cookie tests in younger mice, i.e. when both WT and APP/PS1 mice were 1–2 month-old (Fig. S2). To exclude the possibility that the altered cookie-finding behavior in APP/PS1 mice might be a consequence of impaired locomotion [39], an OFT was performed, with the results showing a slight increase in total distance moved, leaving central distance, time spent in the center and average speed unchanged (Fig. S3a-e). We further performed a typical eight-arm radial maze test using SWSh assessment to evaluate working memory (Fig. S3f). The results showed that APP/PS1 mice at 3–5 months had normal working memory compared to WT controls (Fig. S3g-h).

Interestingly, 3xTg mice [33] also showed impaired cookie-finding and buried-cookie behavior at 3–5 month-old (Fig. 1g-h). The results therefore suggest that abnormal olfaction may been a common defect in AD mouse models, and 3–5 months is the earliest age at which AD mice exhibit impaired odor identification, and we then focused on 3–5 month-old AD animals and WT controls in the following studies unless otherwise indicated.

### Aberrant in vivo theta and gamma oscillations in the OB

**Fig. 2:**
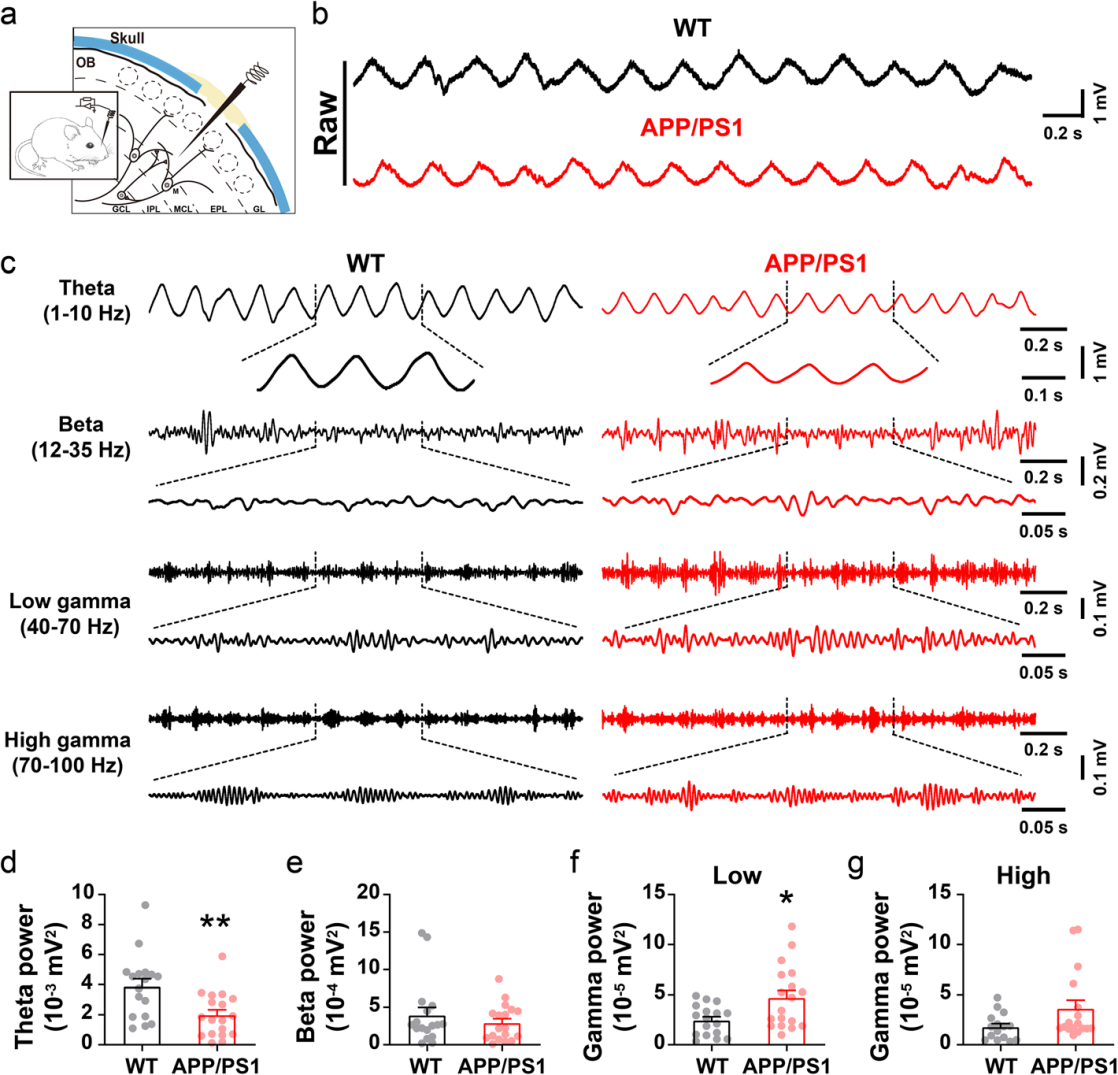
Altered oscillatory activities in the OB of APP/PS1 mice. **a** Schematic diagram showing in vivo LFP recording in the OB. **b**-**c** Representative traces of extracellular recordings showing theta, beta and gamma oscillations in the OB of 3–5 month-old WT and APP/PS1 mice. **d** Oscillatory power in the theta band is significantly reduced in the OB of APP/PS1 mice compared to WT controls (*p* = 0.004). **e** Identical power in the beta band of WT and APP/PS1 mice (*p* = 0.386). **f** Significantly increased power in the low-gamma band (*p* = 0.011). **g** High-gamma power did not differ significantly in APP/PS1 mice compared to WT controls (*p* = 0.06) (*n* = 17 for WT and 18 for APP/PS1). Values represent mean ± SEM. Two-sample t-test. *p < 0.05, **p < 0.01

Neural oscillations, one of the most salient features of in vivo mammalian electrophysiology, can be observed in the OB [27, 51]. To examine whether abnormal oscillatory activity might play a role in impaired olfactory detection, we conducted in vivo extracellular recording around the mitral cell layer (MCL) of the OB in urethane-anesthetized APP/PS1 mice and WT controls (Fig. 2a-c). We observed significantly reduced power in the theta (1–10 Hz) band of APP/PS1 mice compared to WT (Fig. 2d), while beta oscillations remained unchanged (Fig. 2e). Since theta oscillations in the OB are determined by the strength of OSN → OB glutamatergic projections [52, 53], a reduction in theta oscillations may implicate weaker projections from the OSNs of the OE to the OB in APP/PS1 mice.

Gamma oscillations (40–100 Hz) power and periodicity vary with both OSN → OB projections and the degree to which M/T cells couple with the population rhythm of inhibitory neurons, reflecting local circuit interactions within the OB [31, 54]. We observed significantly increased power in the low-frequency gamma band (40–70 Hz) in APP/PS1 mice (Fig. 2f-g), suggesting that circuit activity might be disrupted in the OB of APP/PS1 mice. It is worth noting that, in younger (1–2 month-old) mice, there were no significant differences in these oscillatory activities in the OB between APP/PS1 and WT animals (Fig. S4), in line with normal olfactory behavior in APP/PS1 mice at this age. The results indicate that altered in vivo oscillatory activity in the OB may be an electrical signature of abnormal olfactory behavior in 3–5 month-old APP/PS1 mice.

### Altered miniature excitatory and inhibitory responses of M/T cells

**Fig. 3:**
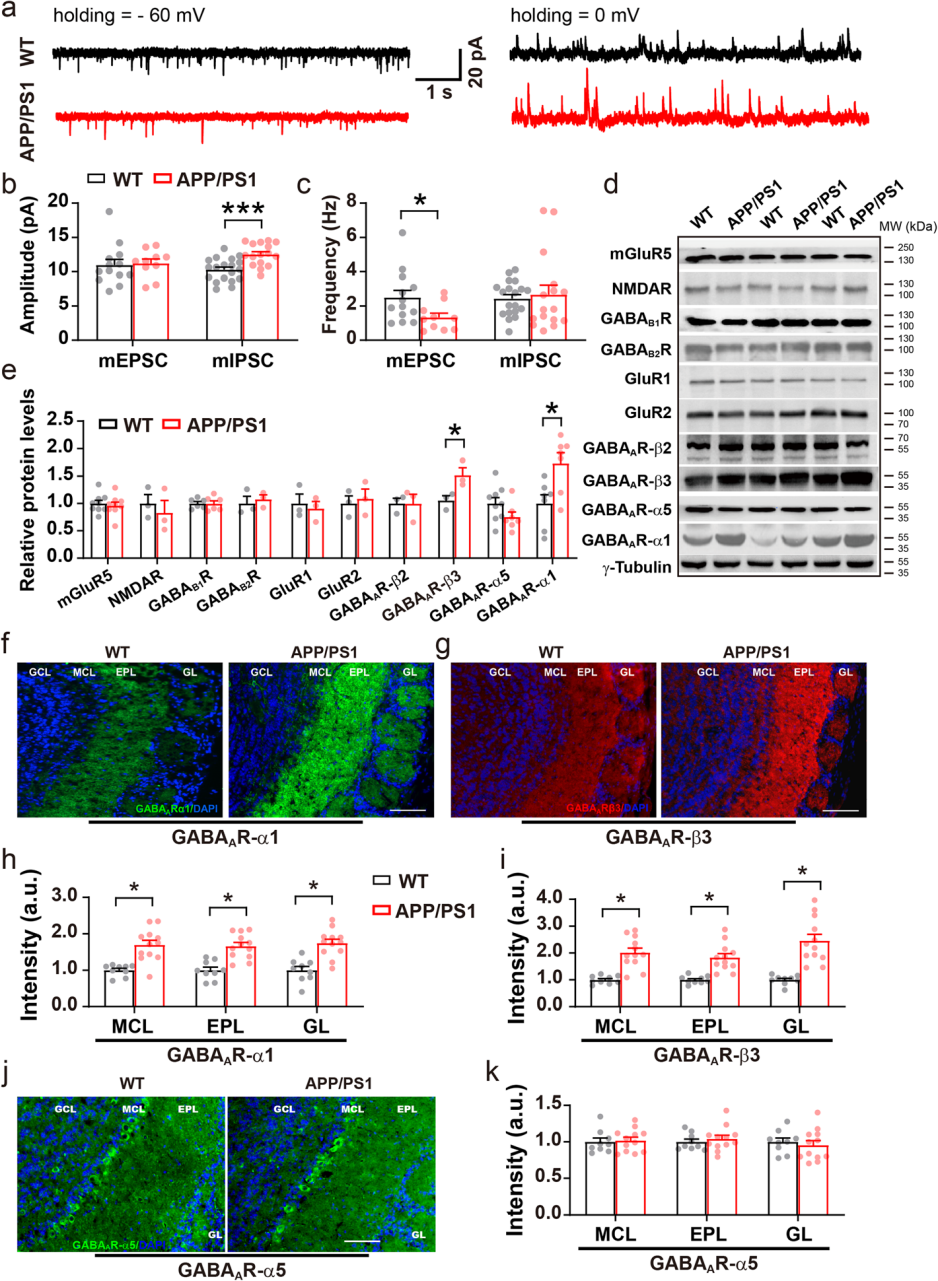
Altered miniature synaptic responses and levels of GABA_A_R subunits in the OB of APP/PS1 mice. **a** Representative mEPSC and mIPSC traces. **b** Significantly increased mIPSC amplitude in the OB of APP/PS1 compared to WT mice (*n* = 19 cells from 3 WT mice and 16 cells from 3 APP/PS1 mice, *p* = 0.0003). **c** Significantly decreased mEPSC frequency in the OB of APP/PS1 compared to WT mice (*n* = 13 cells from 3 WT mice and *n* = 10 cells from 3 APP/PS1 mice, *p* = 0.03). **d**-**e** Representative immunoblots and quantification of excitation- and inhibition-related protein levels in the OB (*n* = 3–9 per genotype, GABA_A_R β3: *p* = 0.03; GABA_A_R α1: *p* = 0.03). **f**-**i** Representative images and statistical analysis of immunofluorescent staining of GABA_A_R α1 and β3 subunits in the OB of WT and APP/PS1 mice at 3–5 months (*n* = 3 slices/mouse, 3 mice for WT and 4 mice for APP/PS1; GABA_A_Rα1: *p* = 0.036 for MCL, *p* = 0.032 for EPL, *p* = 0.021 for GL; GABA_A_Rβ3: *p* = 0.021 for MCL, *p* = 0.019 for EPL, *p* = 0.033 for GL). **j**-**k** Representative images and statistical analysis of immunofluorescent staining of GABA_A_R α5 subunit (*n* = 3 sections/mouse, 3 mice for WT and 4 mice for APP/PS1; *p* = 0.793 for MCL, *p* = 0.529 for EPL, *p* = 0.597 for GL). Scale bar: 100 μm. Values represent mean ± SEM. Two-sample t-test. *p < 0.05; ***p < 0.001

Disturbance of the E/I ratio results in altered gamma oscillations in the OB [27]. OSN axons terminate in the glomerular layer (GL) of the OB releasing the excitatory neurotransmitter, glutamate, onto M/T cells [27, 55]. The latter synapses with GABAergic neurons, such as granule cells (GCs) [27, 56], parvalbumin interneurons (PV) [57, 58] and short axon cells [59], providing broad feedback control which is critical for oscillatory activity [27, 60, 61]. To examine whether abnormal gamma oscillations in the OB involves altered OSN → M/T cell excitation and M/T cell inhibition in APP/PS1 mice, we next performed whole-cell patch-clamp recording in the M/T cells of acute OB slices (Fig. 3a). These experiments revealed similar amplitude but a significantly reduced frequency of the mEPSC in M/T cells (Fig. 3b-c, WT: 2.5 ± 0.4 Hz, APP/PS1: 1.3 ± 0.2 Hz). Together with the LFP result showing reduced theta power, these results suggest that excitatory synaptic transmission in the OSN → M/T pathway is abnormal, presumably due to presynaptic excitatory deficits. At the same time, the amplitude of the mIPSC in M/T cells increased significantly (Fig. 3b, WT: 10.3 ± 0.4 pA, APP/PS1: 12.5 ± 0.4 pA), leaving the frequency of mIPSC unchanged, implicating an existence of postsynaptic GABAergic alteration.

### Upregulation of the GABA_A_R subunits co-occurs with increased mIPSC amplitude

GABAergic mechanisms, particularly GABA_A_R-mediated inhibition, contribute greatly to the generation of gamma oscillations [62, 63] and mIPSCs [64, 65] in M/T cells. Glutamate release from M/T cells, which triggers recurrent excitation via AMPA (AMPARs) and NMDA receptors (NMDARs), also supports oscillatory activities [27]. We next used western blotting to measure the protein levels of both excitatory and inhibitory neurotransmitter receptors in the OB. Consistent with the increased mIPSC amplitude noted above, we observed significantly increased levels of the GABA_A_R α1 and β3 subunit. Levels of other GABA_A_R subunits were unchanged (Fig. 3d-e, Additional file 1). In contrast, levels of G protein-coupled GABA_B_Rs and subunits of the excitatory AMPARs and NMDARs were similar between WT and APP/PS1 mice (Fig. 3d-e, Additional file 1). Furthermore, immunofluorescent staining confirmed the increased expression of GABA_A_R α1 and β3 subunits (Fig. 3f-i) with distribution patterns as reported [64, 66]. Given the M/T cell-specific expression of GABA_A_R α1 subunit [64, 67], altered levels of GABA_A_R subunits reveal an abnormal postsynaptic inhibitory response in the OB of APP/PS1 mice. In agreement with western blotting analysis, immunofluorescent staining of GABA_A_R α5 subunit remained unchanged (Fig. 3j-k).

### Reduction in PPR of IPSC indicates abnormal presynaptic probability of GABA release

**Fig. 4:**
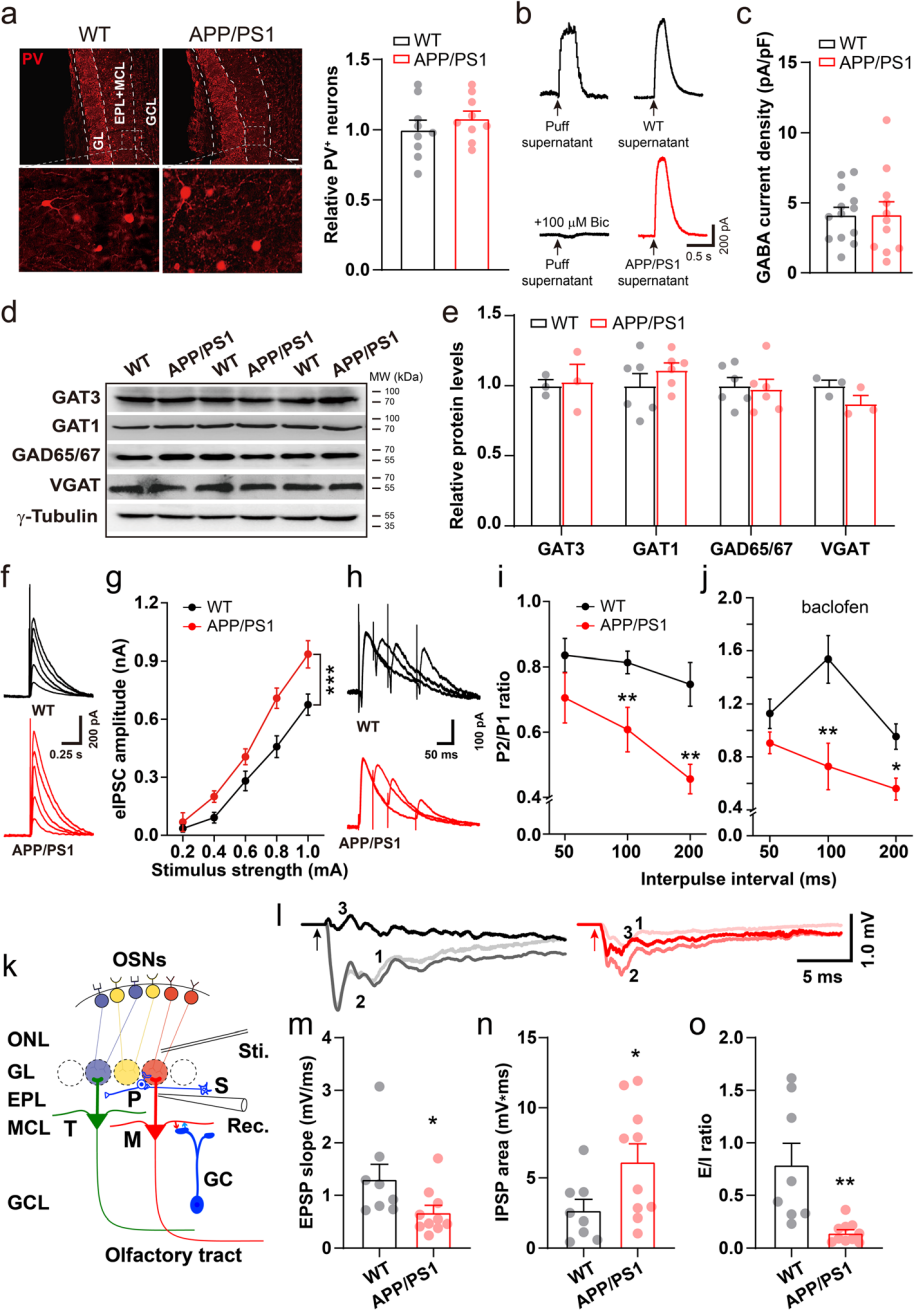
Abnormal PPR and field IPSP in APP/PS1 mice. **a** Identical numbers of PV-positive interneurons in the OB of APP/PS1 and WT mice (*n* = 3 slices/mouse, 3 mice for each genotype). **b** Representative traces showing evoked GABA current of WT mice in response to puffing brain lysates obtained from WT and APP/PS1 OB, respectively. Each trace represents an average of five sweeps. **c** Quantification showing similar GABA current density induced by puffing OB supernatant of APP/PS1 and WT mice, indicating total GABA content in the OB did not differ significantly between the two genotypes (*n* = 13 cells for WT-, and *n* = 11 for APP/PS1-supernatants). **d**-**e** Representative immunoblots and quantification of several GABAergic transporters in the OB. **f**-**g** Sample traces of whole-cell patch recording of single eIPSC and quantification showing a significant increase in the amplitude of eIPSCs in APP/PS1 mice (*n* = 8 cells from 3 WT and 9 cells from 3 APP/PS1, *p* = 3 × 10^− 7^ for interaction of genotype and stimulus strength, two-way ANOVA with Bonferroni’s post-hoc test). **h**-**i** Representative traces and quantification of paired-pulse responses. PPR decreased significantly at 100- and 200-ms interpulse intervals (*n* = 8 cells from 3 WT and 9 cells from 3 APP/PS1; 100 ms: *p* = 0.004; 200 ms: *p* = 0.004, one-way ANOVA with Tukey’s post-hoc test). **j** Similar effect of baclofen on PPR of both groups (*n* = 11 cells from 5 WT and 8 cells from 3 APP/PS1; 50 ms: *p* = 0.157; 100 ms: *p* = 0.006; 200 ms: *p* = 0.011, one-way ANOVA with Tukey’s post-hoc test). **k** Diagram showing field EPSP recording in slice. P: periglomerular cell, S: short axon cell. **l** Representative traces of ONL stimulation induced mixed EPSP (indicated as 1), pure EPSP (2) and IPSP (3). (**m-o**) Significantly reduced slope of pure EPSP (*n* = 8 slices from 4 WT and 10 slices from 4 APP/PS1, *p* = 0.045, two-sample t-test), increased area of IPSP (*p* = 0.044, two-sample t-test) and reduced E/I ratio (*p* = 0.003, nonparametric Kolmogorov-Smirnov test). Values represent mean ± SEM. *p < 0.05; **p < 0.01; ***p < 0.001

Fast-spiking PV neuron, one-type GABAergic interneurons in the OB, are dominant presynaptic partners of mitral cells [68]. PV neurons form reciprocal connections with the majority of nearby mitral cells, mediating inhibition onto mitral cells [57] and play an important role in gamma oscillations [58, 69, 70]. We therefore determined whether the abnormal gamma oscillations observed in the OB involved altered numbers of GABAergic PV interneurons. However, immunofluorescent staining showed that numbers of PV-positive neurons in WT and APP/PS1 OB were similar (Fig. 4a). Next, GABA content in the OB was evaluated by puff-protocol, a method for evaluating the amount of GABA in the tissue collected [42]. In the presence of APV and CNQX to block excitatory responses, postsynaptic current induced by OB supernatant-puff could be blocked by a GABA_A_R antagonist, bicuculline, suggesting supernatant evoked IPSC (Fig. 4b, left panel). Next, the amplitude of IPSCs induced by supernatant-puff obtained from WT or APP/PS1 OB was similar (Fig. 4b, right panel, c, WT: 4.2 ± 0.5 pA/pF, APP/PS1: 4.2 ± 0.9 pA/pF), suggesting identical levels of GABA in the OB of the two groups. Furthermore, ELISA analysis revealed unchanged amount of GABA in APP/PS1 OB compared to WT controls (WT: 8.13 ± 0.34 ng/ml, APP/PS1: 8.44 ± 0.61 ng/ml; *p* = 0.67, *n* = 6 mice for each genotype). Together, these results suggest that overall GABA content, a resource of GABA transmitter, was similar in the OB of the two groups.

Levels of coherent transporters, such as GAT1, GAT3, VGAT and GAD65/67, which are critical for GABAergic inhibitory activities, are important in regulating the concentration of GABA in the extracellular matrix [71]. However, immunoblotting analysis revealed that the levels of the above proteins were unchanged in APP/PS1 OB (Fig. 4d-e, Additional file 1).

Analysis of mIPSCs revealed a baseline postsynaptic alteration in the inhibitory response of M/T cells. We next measured the evoked inhibitory response of acute OB slices in the presence of CNQX and APV to block the excitatory response. The whole-cell IPSC of M/T cells was induced by single or double electrical stimulation of the area containing inhibitory axonal terminals, and the amplitudes of single and paired-pulse evoked monosynaptic IPSCs (eIPSC) were analyzed. Consistent with the increased mIPSC amplitude in the M/T cells of APP/PS1 mice, the eIPSC amplitude increased significantly in APP/PS1 OB compared to WT controls (Fig. 4f-g), again indicating a postsynaptic alteration due, likely, to upregulation of GABA_A_R subunits. Alternatively, such an increase in the eIPSC amplitude of M/T cells may also involve a higher presynaptic probability of release of GABA during the initial response, which commonly results in a reduced probability of release following a second stimulation, i.e., a reduction in the PPR (given by IPSC2 amplitude/IPSC1 amplitude) [72, 73]. We next conducted a paired-pulse protocol that has been widely used to evaluate presynaptic function [42, 74] in OB slices. A significantly decreased PPR was observed at 100- and 200-ms interpulse intervals (Fig. 4h-i, 50 ms: WT, 0.83 ± 0.05, APP/PS1, 0.69 ± 0.06; 100 ms: WT, 0.82 ± 0.03, APP/PS1, 0.61 ± 0.05; 200 ms: WT, 0.73 ± 0.06, APP/PS1, 0.49 ± 0.04), indicating an increased GABA release probability in APP/PS1 mice.

GABA_B_Rs are involved in presynaptic modulation of dendrodendritic signaling between GC and M/T cells [75]. The selective GABA_B_R agonist, baclofen, reduces release probability by inhibiting presynaptic GABA release [76], leading to subsequent increase in PPR. We thus determined whether or not baclofen differentially regulated PPR in WT and APP/PS1 mice. The results showed that baclofen similarly increased PPR in the two groups, leaving the significant difference of PPR, at 100- and 200-ms interpulse intervals, unchanged (Fig. 4j) (100 ms: WT, 1.5 ± 0.18, APP/PS1, 0.73 ± 0.18; 200 ms: WT, 0.95 ± 0.10, APP/PS1, 0.56 ± 0.08). The PPR result was in line with identical levels of GABA_B_Rs between the two groups (Fig. 3d-e). Thus, aberrant PPR may result from altered release kinetics rather than abnormality in levels of GABA and GABA_B_Rs mediated GABA release.

### Dissection of recurrent inhibition from the total response of the OE → OB circuit

The OSNs in the OE are the glutamatergic upstream sources that innervate M/T cells [27, 55]. Thus, a reduction in mEPSC frequency in the M/T cells might suggest possible presynaptic glutamatergic defects in the OSN → M/T pathway, which could cause impairment in M/T cell excitation and subsequent inhibition. To evaluate the function of OE → OB neural circuitry, the axonal terminals of OSNs (ONLs) were stimulated and the field excitatory postsynaptic potential (EPSP) was recorded in the external plexiform layer (EPL) of the dendritic area of M/T cells in horizontal OB slices (Fig. 4k). EPSPs recorded in the presence or absence of a GABA receptor antagonist, Picrotoxin, represent pure (Fig. 4l, 2) and mixed EPSPs (Fig. 4l, 1), respectively. We observed a significantly reduced slope of pure EPSPs, indicating a reduction in the OSN-induced excitatory response of M/T cells in APP/PS1 mice (Fig. 4m, WT, 1.31 ± 0.26 mV/ms, APP/PS1, 0.67 ± 0.13 mV/ms).

Given that OSN → M/T cell excitation activates GABAergic interneurons, which in turn induce inhibition of M/T cells [27, 59, 68], we next evaluated the Picrotoxin-sensitive component, which represents inhibitory postsynaptic potential (IPSP), by subtracting the area of mixed EPSP from pure EPSP (Fig. 4l, 3). We found that the IPSP area increased significantly in APP/PS1 mice, suggesting that ONL stimulation induced a stronger inhibitory response in M/T cells (Fig. 4n, WT, 2.69 ± 0.73 mV*ms, APP/PS1, 6.15 ± 1.21 mV*ms). A decreased EPSP and an increased IPSP resulted in a significant reduction in E/I ratio, as determined by EPSP slope/IPSP area (Fig. 4o, WT, 0.79 ± 0.19, APP/PS1, 0.15 ± 0.03). Given that a reduction in ONL-evoked EPSP indicates a weaker ability of M/T cells to trigger GCs and other interneurons releasing GABA, an increased IPSP in M/T cells may be attributed to compensatorily upregulated presynaptic release probability and levels of GABA_A_R α1 and β3 subunits in APP/PS1 mice.

### Reduction of EOG amplitude and number of mature OSNs

**Fig. 5:**
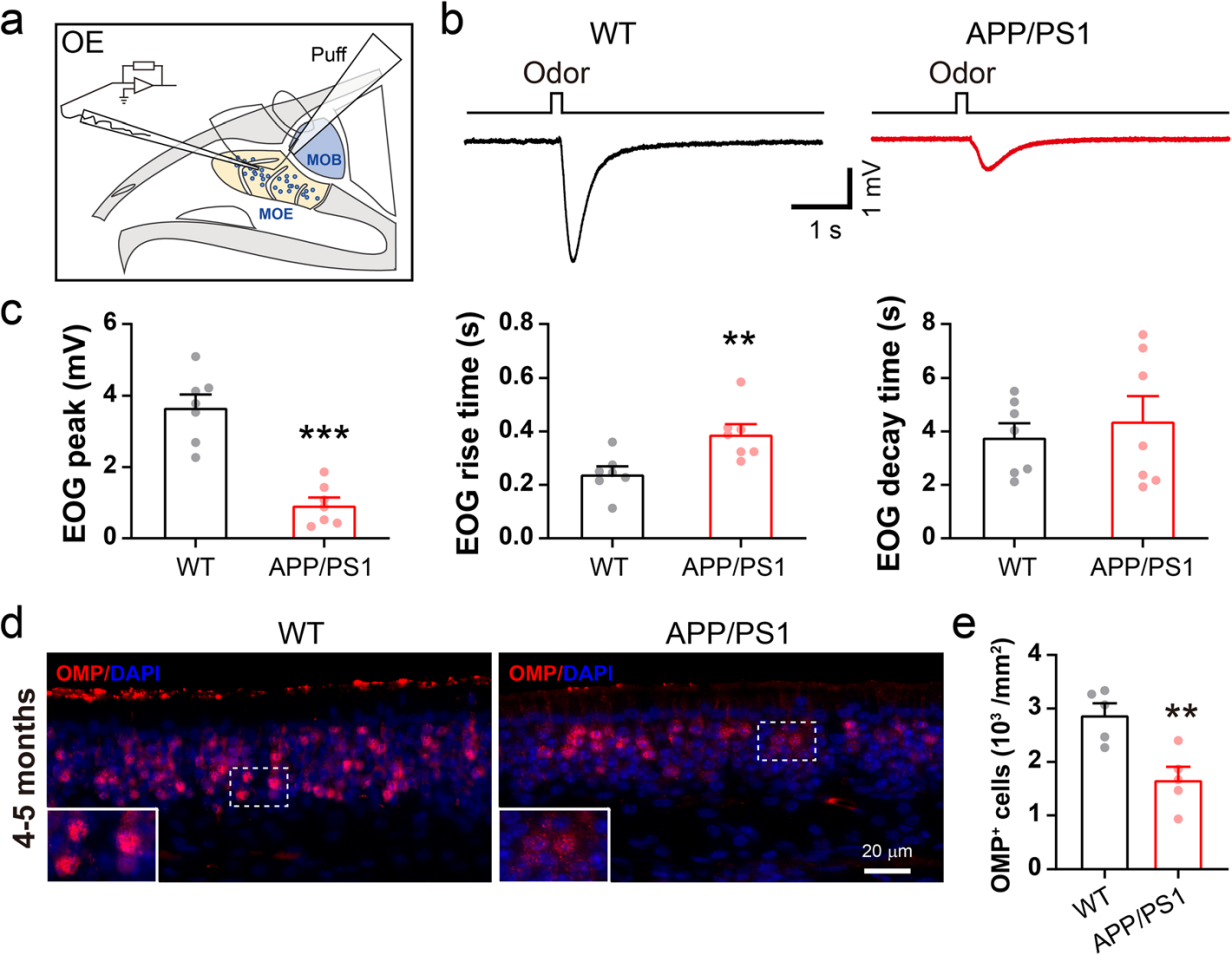
Reduction in the EOG and numbers of mature OSNs in APP/PS1 OE. **a** Diagram showing EOG recording in the olfactory turbinate. **b** Representative EOG traces from olfactory turbinate of WT and APP/PS1 mice in response to odor (amyl acetate) application. **c** Significantly decreased EOG amplitude (*p* = 3 × 10^− 5^), increased rise time (*p* = 0.008) and unchanged decay time of EOG (*p* = 0.583) (*n* = 7, per genotype). **d**-**e** Immunofluorescent staining of OMP and quantification showing marked reduction in numbers of mature OSNs in the APP/PS1 OE (*n* = 5 slices from 3 mice, per genotype, *p* = 0.005). Scale bar: 20 μm. Values represent mean ± SEM. Two-sample t-test. **p < 0.01; ***p < 0.001

The OSNs and their axonal terminals are presynaptic excitatory compartment of M/T cells. To further examine the presynaptic mechanism underlying impaired M/T cell excitation, we recorded odor-evoked in vitro EOGs, which reflect the sum of the generator potentials of mature OSNs in the OE, in the medial part of the OE of the olfactory turbinate (Fig. 5a) as described [34, 77]. We found that the average EOG peak amplitude induced by puff application of amyl acetate decreased significantly in 3–5 month-old APP/PS1 mice compared to WT controls (Fig. 5b-c, WT: 3.7 ± 0.4 mV; APP/PS1: 0.9 ± 0.2 mV). In addition, the relative rise time increased in 3–5 month-old APP/PS1 mice (Fig. 5c, WT: 0.24 ± 0.03 s; APP/PS1: 0.39 ± 0.04 s), leaving the relative decay time unchanged. These results suggest that 3–5 month-old APP/PS1 mice have a weaker odor-evoked response in the OE than controls.

Deficits in odor identification are among the earliest symptoms of AD patients [24, 78] and loss of mature OSNs could contribute to this pathology [79]. To determine whether an altered number of OSNs underlies the aberrant EOGs in the OE of APP/PS1 mice, we next performed immunofluorescent staining using an antibody against the olfactory marker protein (OMP) of mature OSNs. We observed significantly reduced levels of OMP in APP/PS1 mice (Fig. 5d-e), indicating that a loss of mature OSNs may contribute directly to the abnormal EOGs observed. The axonal terminals of OSNs in the GL of the OB form the first glutamatergic synapse of the main olfactory pathway at the OB surface [80, 81]. Thus, a reduction in the number of mature OSNs would result in less glutamatergic innervation of M/T cells, leading to reduced excitation and theta oscillations seen in the present study [52, 53], in addition to decreased EOG amplitude. It is worth noting that both EOG and number of OSN remained similar in 1–2 month-old APP/PS1 and WT mice (Fig. S5).

### TGB attenuates upregulated gamma oscillations and levels of GABA_A_R

**Fig. 6:**
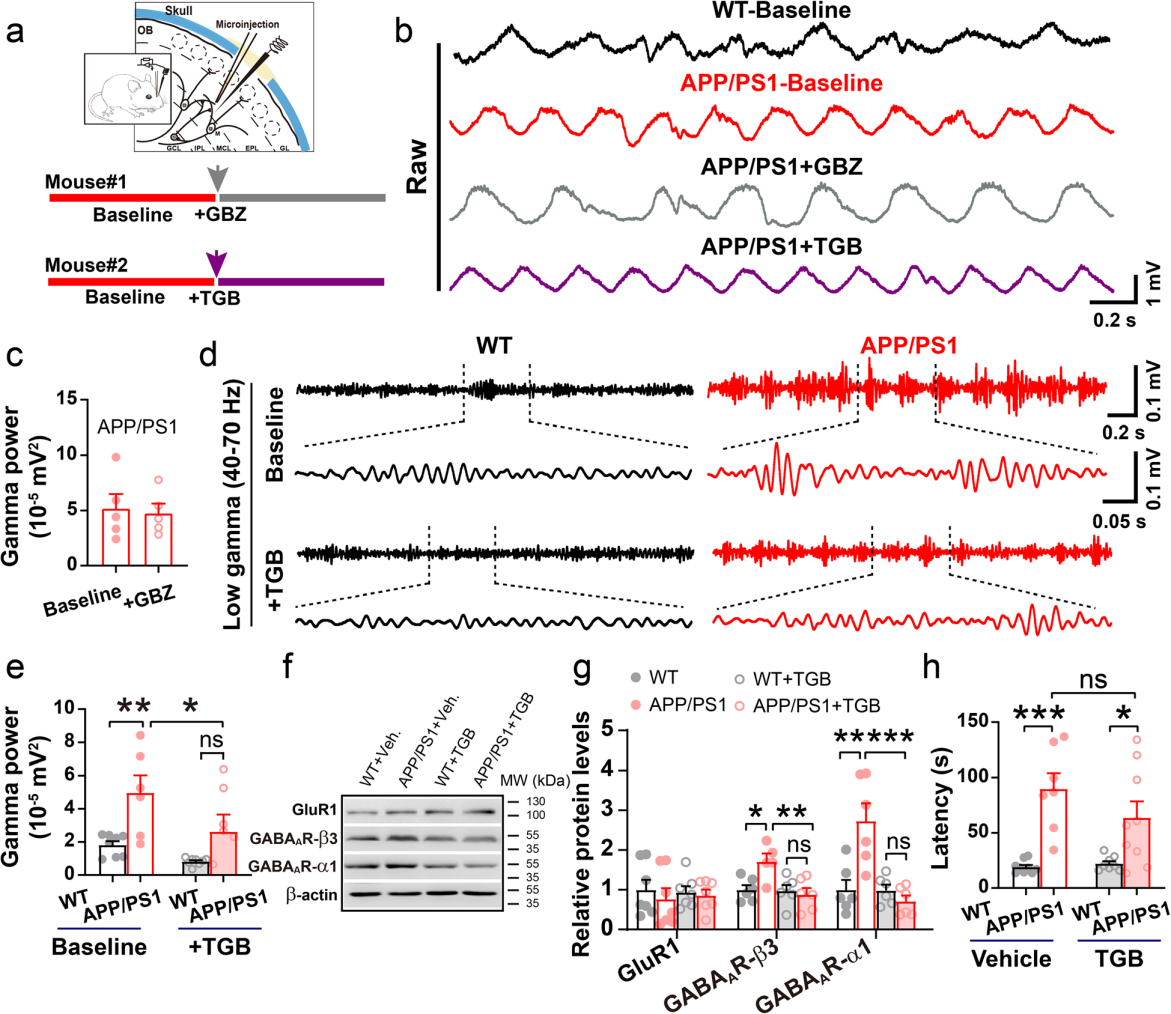
TGB attenuates aberrant gamma power and levels of GABA_A_Rs. **a** Diagram illustrating simultaneous in vivo microinjection and LFP recording in the OB. **b**-**c** Representative traces and quantification of gamma oscillations in APP/PS1 OB before (baseline) and after microinjection of GBZ at 500 μM. GBZ injection has no significant effect on gamma power (*n* = 5 mice, *p* = 0.33, paired sample t-test). **d** Representative traces of gamma oscillation in WT and APP/PS1 before and after microinjection of TGB. **e** Increased gamma oscillatory power in APP/PS1 OB is ameliorated after acute TGB microinjection compared to baseline (*n* = 9 for WT and *n* = 6 for APP/PS1, *p* = 0.002 for WT vs APP/PS1; *p* = 0.02 for APP/PS1 baseline vs APP/PS1 + TGB; *p* = 0.06 for WT + TGB vs APP/PS1 + TGB, two-way ANOVA with Bonferroni’s post-hoc test). **f**-**g** Representative immunoblots and quantification of GABA_A_R α1, β3 subunits and GluR1 in the OB of TGB- and vehicle-treated mice. TGB significantly reduced levels of GABA_A_R α1 and β3 subunits (*n* = 5–8 mice/group, GABA_A_R β3: *p* = 0.03 for WT vs APP/PS1; *p* = 0.009 for APP/PS1 vs APP/PS1 + TGB; GABA_A_R α1: *p* = 0.001 for WT vs APP/PS1; *p* = 0.0002 for APP/PS1 vs APP/PS1 + TGB, two-sample t-test). **h** One-week TGB did not improve olfactory behavior in APP/PS1 mice (*n* = 7–9 mice/group; *p* = 0.0002 for WT + PBS vs APP/PS1 + PBS; *p* = 0.037 for WT + TGB vs APP/PS1 + TGB; *p* = 0.52 for APP/PS1 + PBS vs APP/PS1 + TGB, two-sample t-test). Values represent mean ± SEM. *p < 0.05; **p < 0.01; ***p < 0.001

Generating gamma oscillations involves fast GABAergic transmission mediated by the GABA_A_R and microinfusion of a GABA_A_R antagonist, GBZ, increases gamma power within 30–50 min post microinfusion [27, 82]. To determine whether or not upregulated levels of GABA_A_R in the OB mediate the increase in gamma power, following baseline recording, GBZ was injected locally **(**Fig. 6a-b). However, microinjection of 2–4 μl GBZ at 10 μM or 20 μM (data not shown), and even as high as 500 μM (Fig. 6c), showed no effect on gamma oscillations in APP/PS1 OB. Given that gamma power is significantly increased in APP/PS1 mice, perhaps this increase has a saturation point; thus, GBZ cannot further increase gamma power in APP/PS1 mice.

Given that GABAergic inhibition was, likely, compensatorily enhanced in the OB of APP/PS1, we next tested whether enhancement of GABAergic inhibition by microinjection of TGB, an inhibitor of GABA transporter GAT1 that increases levels of GABA in the synaptic cleft [43, 82], could ameliorate abnormal gamma-oscillations. Interestingly, microinjection of TGB did indeed attenuate gamma power in some of the APP/PS1 mice and the average gamma power no longer differed significantly between TGB-treated APP/PS1 and WT controls (Fig. 6d-e). Moreover, we found that daily IP injections of TGB over a period of 1 week significantly reduced levels of GABA_A_R α1 and β3 subunits in the OB of APP/PS1, leaving levels of GluR1 unchanged (Fig. 6f-g, Additional file 1). In addition, the APP/PS1 mice were still spending a longer time in the cookie test than WT in the same experiment where they were administered TGB (Fig. 6h). This suggests that abnormal olfaction might be a manifestation correlating to aberrant gamma oscillations at the time point tested, but not a direct consequence of altered gamma wave or alternatively, that acute and short-term amelioration of gamma oscillations may not be sufficient to improve olfaction.

### Altered connectivity between OB and PC

**Fig. 7:**
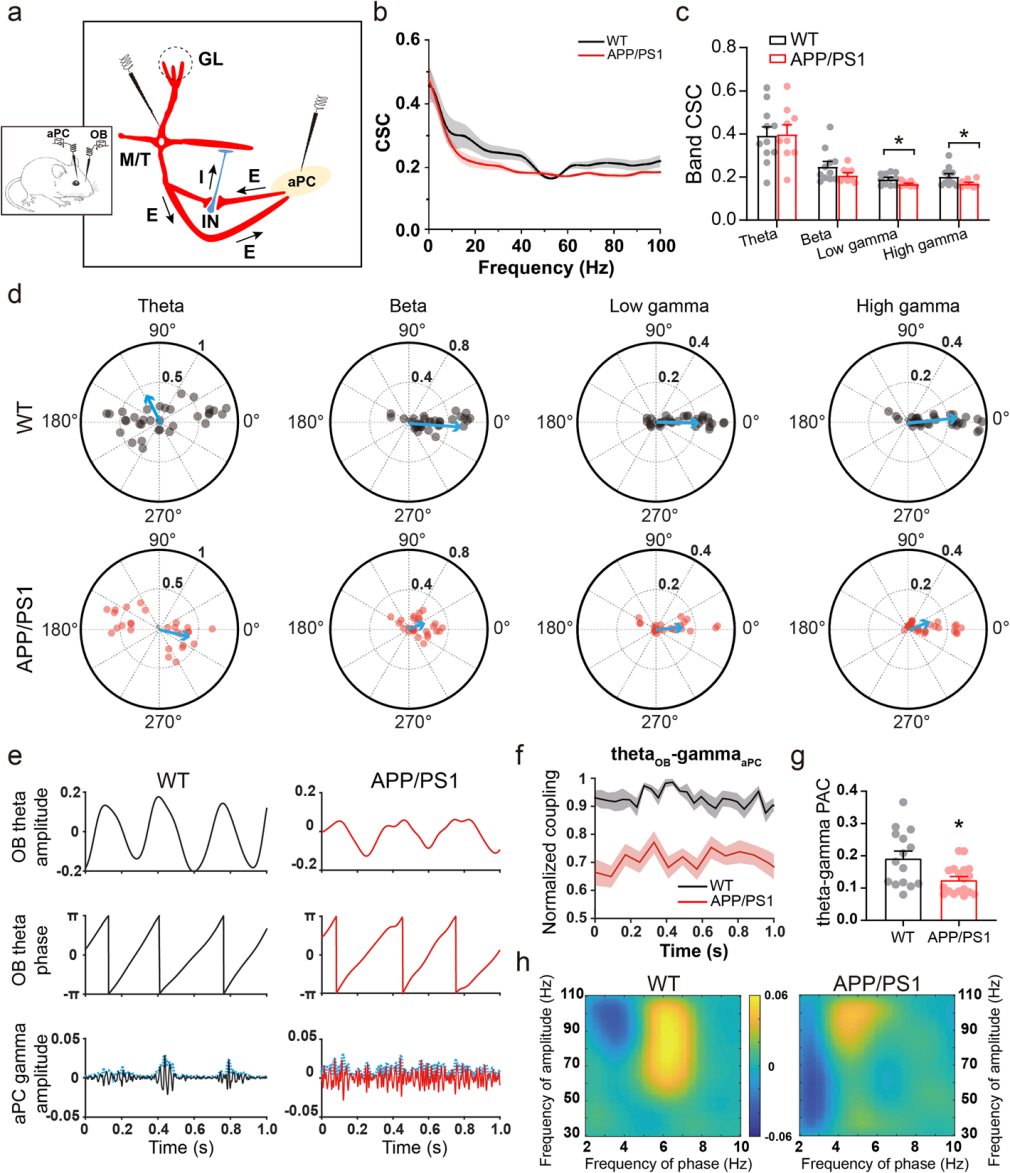
Altered synchronization and coupling between OB and aPC in APP/PS1 mice. **a** Schematic diagram showing in vivo dual-site extracellular recordings in the OB and aPC. **b**-**c** Cross coherence of simultaneous LFPs in the OB and aPC indicating significantly decreased consistency between OB and aPC in both low-gamma and high-gamma bands in APP/PS1 mice (*n* = 11 for WT and 9 for APP/PS1; theta: *p* = 0.761; beta: *p* = 0.197; low gamma: *p* = 0.04; high gamma: *p* = 0.024, Wilcoxon rank-sum test). **d** Significantly shorter mean vector length of PSC in beta (*p* = 1.51 × 10^− 10^) and high gamma (*p* = 0.034, Wilcoxon rank-sum test) phases, and significantly different distributions of phase differences showing decreased phase synchronization between OB and aPC. The resultant vector across animals is shown as blue arrows and values represent individual PSCs of identical frequency band. **e** Typical examples of instantaneous phases derived from OB theta oscillations and aPC gamma oscillations’ amplitude, which were used for cross frequency PAC calculation. **f** Dynamic changing curves of 1-s interval PAC in both genotypes. Note that APP/PS1 differed largely from WT in normalized units. **g** Quantification showing significantly decreased theta-gamma cross-band PAC between the OB and aPC in APP/PS1 mice (*n* = 15 epochs from 11 WT and 18 epochs from 9 APP/PS1, *p* = 0.016, Wilcoxon rank-sum test). **h** APP/PS1 displayed less strength of information flow than WT mice. Directionality confirmed PAC mainly from the OB to aPC with more positive values. Values represent mean ± SEM. *p < 0.05

The output of encoded odor information by M/T cells is sent to a variety of higher brain regions, including the aPC [83, 84], which in turn directly innervates GABAergic interneurons of the OB via a long-range cortical feedback excitatory projection [85, 86]. This loop is also thought to contribute to oscillatory dynamics in the OB [27, 87]. We next evaluated OB-PC connectivity by simultaneous LFP recording in the OB and aPC (Fig. 7a). Firstly, we found that CSC between LFPs of the two areas significantly reduced at gamma band in APP/PS1 mice (Fig. 7b-c, theta, WT: 0.394 ± 0.040, APP/PS1: 0.400 ± 0.043; beta, WT: 0.250 ± 0.023, APP/PS1: 0.210 ± 0.011; low gamma, WT: 0.191 ± 0.008, APP/PS1: 0.169 ± 0.004; high gamma, WT: 0.203 ± 0.013, APP/PS1: 0.170 ± 0.006).

Considering that the calculation of coherence might be disturbed by the amplitude of the LFP signals on synchrony, we next used PSC, which is relatively insensitive to power effects and could reveal the synchronous level of oscillations between the OB and aPC in more precise phase ranges [48, 88]. The PSC analysis confirmed that APP/PS1 mice had less interregional synchrony with significantly shorter mean vector length and differently distributed phase differences in the beta and gamma bands (Fig. 7d, beta, WT: 0.453 ± 0.02, APP/PS1: 0.184 ± 0.01; high gamma, WT: 0.264 ± 0.03, APP/PS1: 0.157 ± 0.02). Together, the above results suggest impaired neuronal connectivity between the aPC and the OB in APP/PS1 mice.

Theta–gamma PAC, in which the amplitude of gamma rhythms is nested to the phase of theta rhythms, reflects the spatial-temporal pattern of information processing within specific neural circuits [49, 89]. Therefore, we further analyzed how the aPC gamma activity that has high enough oscillatory amplitude been coupled with OB theta oscillations (Fig. 7e-f), and observed significantly reduced theta-gamma PAC in APP/PS1 mice (Fig. 7g, WT: 0.192 ± 0.02, APP/PS1: 0.125 ± 0.01). Furthermore, lower directionality, identified by “colder” maximal OB → aPC PAC values, revealed less OB-aPC coupling in APP/PS1 mice (Fig. 7h), suggesting a reduced strength of information flow from the OB to aPC.

### Amelioration of gamma oscillations by inhibition of Aβ production

**Fig. 8:**
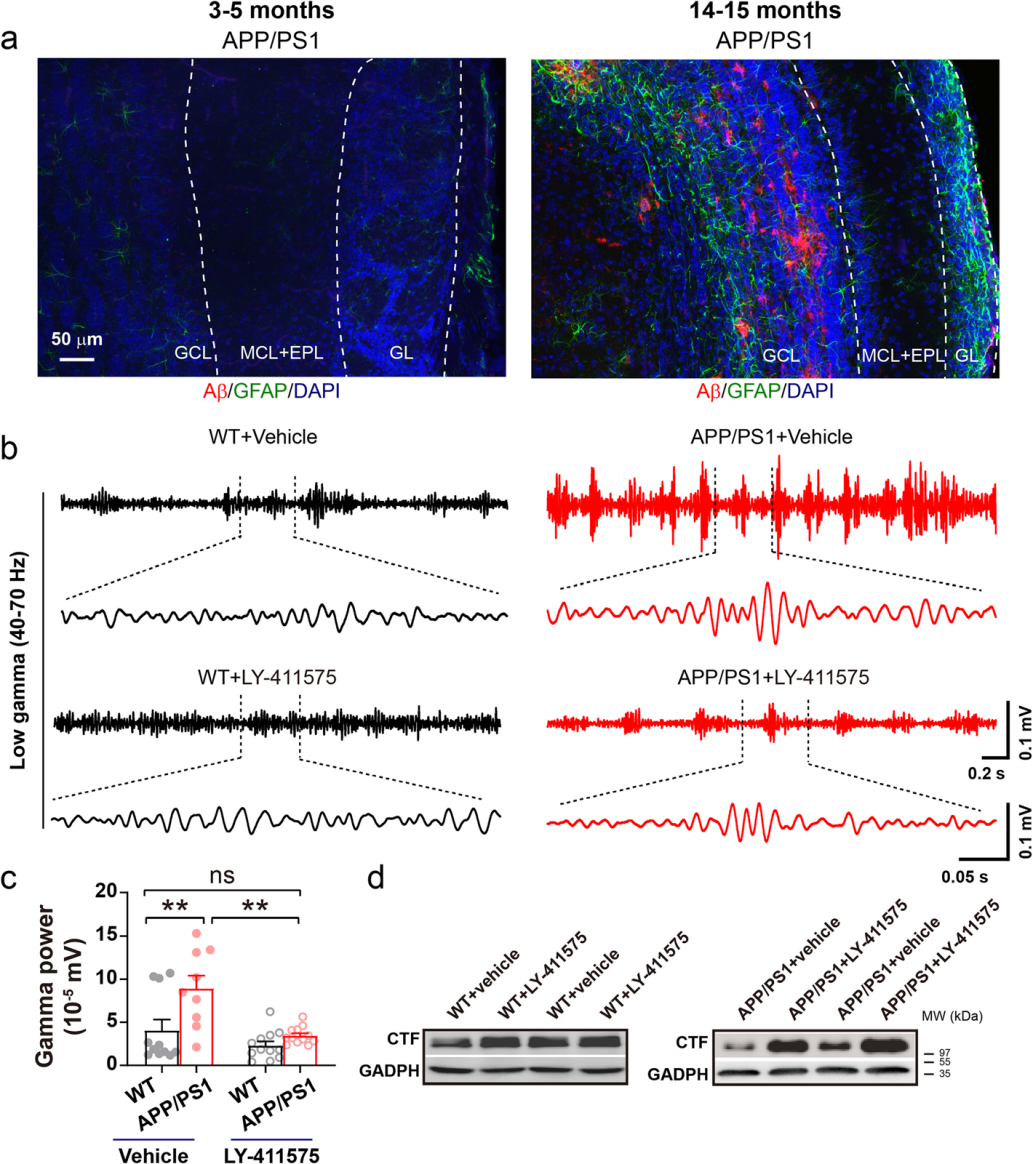
Reducing interstitial fluid levels of Aβ reverses the abnormalities of gamma power in APP/PS1 OB. **a** Representative images of Aβ deposition in the OB of APP/PS1 mice aged 3–5 and 14–15 months, suggesting a lack of obvious amyloid plaques in 3–5 month-old APP/PS1 OB. Scale bar: 50 μm. **b**-**c** Two-day IP injection of LY-411575 significantly decreased gamma power (*n* = 11 for vehicle-treated WT and 12 for LY-411575-treated WT, *n* = 9 for vehicle-treated APP/PS1 and 12 for LY-411575-treated APP/PS1; *p* = 0.006 for WT + vehicle vs APP/PS1 + vehicle; *p* = 0.001 for APP/PS1 + vehicle vs APP/PS1 + LY-411575, two-way ANOVA with Bonferroni’s post-hoc test). **d** Increased levels of CTFs specifically in APP/PS1 OB confirming the effect of LY-411575 in reducing Aβ production. Values represent mean ± SEM. **p < 0.01

Alterations in olfaction and OB neuronal activities have been shown to be associated with increased soluble Aβ levels [21, 23]. Although the OB of 3–5 month-old APP/PS1 mice did not exhibit the Aβ plaques found in 14–15 month-old APP/PS1 mice (Fig. 8a), the high levels of soluble Aβ in this AD model may cause synaptic and network impairment in the OB. We thus conducted IP injections over a two-day period of a γ-secretase inhibitor, LY-411575, which has been shown to reduce soluble Aβ production [45, 90], and observed significantly reduced gamma-oscillation power in the OB of LY-411575-treated APP/PS1 mice (Fig. 8b-c). The effectiveness of LY-411575 in limiting Aβ production in the OB of APP/PS1mice was supported by the presence of elevated levels of C-terminal fragments (CTFs) of APP (Fig. 8d). The results confirm that Aβ overproduction contributes to enhanced gamma oscillations in the OB of APP/PS1 mice.

### 3xTg mice also exhibit aberrant gamma oscillations in the OB

**Fig. 9:**
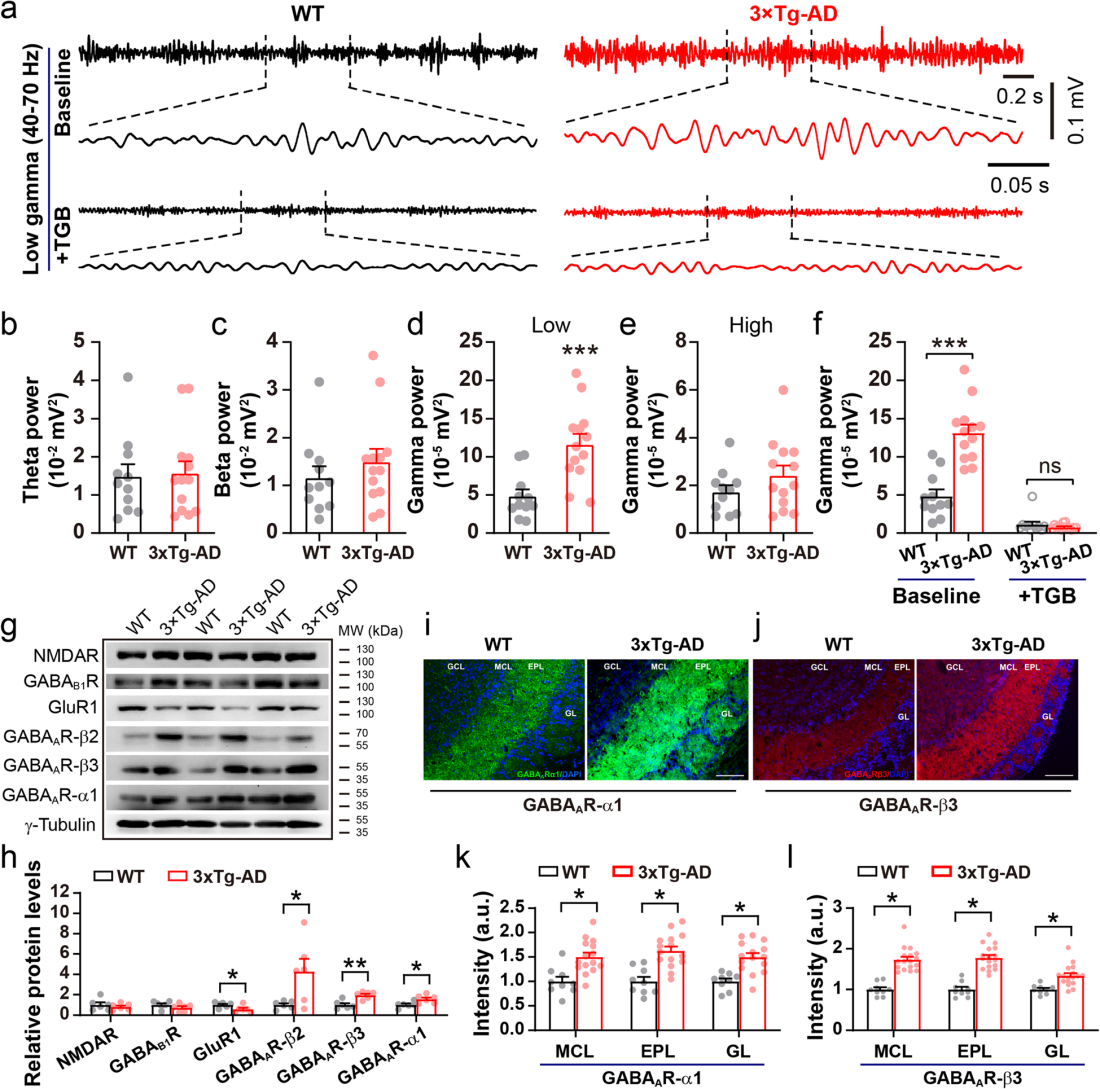
Abnormal oscillatory activities and the effect of TGB in the OB of 3xTg mice. **a** Representative gamma oscillation in the OB of 3–5 month-old WT and 3xTg mice. **b**-**c** Unchanged power in the theta and beta band of 3xTg compared to WT mice. **d**-**e** Significantly increased power in low-gamma band (*p* = 0.0007) and similar power in high-gamma band (*p* = 0.195) of WT and 3xTg mice. **f** TGB significantly reduced gamma power and normalized the difference of gamma oscillation between 3xTg and WT (*n* = 11 for WT and *n* = 13 for 3xTg; *p* = 0.0001, WT vs 3xTg; *p* = 0.0001, 3xTg vs 3xTg + TGB; *p* = 1, WT + TGB vs 3xTg + TGB). **g**-**h** Representative immunoblots and quantification of excitation- and inhibition-related protein levels in the OB (*n* = 3, per genotype, two repeats, GluR1: *p* = 0.032; GABA_A_Rβ2: *p* = 0.026; GABA_A_Rβ3: *p* = 0.001; GABA_A_Rα1: *p* = 0.02). (**i-l**) Representative images and statistical analysis of immunofluorescent staining of GABA_A_R α1 and β3 subunits in the OB of WT and 3xTg mice at 3–5 months (*n* = 3–4 sections/mouse, 3 mice for WT and 5 mice for 3xTg; GABA_A_R α1: *p* = 0.036 for MCL, *p* = 0.032 for EPL, *p* = 0.021 for GL; GABA_A_R β3: *p* = 0.021 for MCL, *p* = 0.019 for EPL, *p* = 0.033 for GL). Values represent mean ± SEM. Two-sample t-test. *p < 0.05; **p < 0.01; *** *p* < 0.001

Finally, to determine whether aberrant gamma oscillations occur in other AD models, we conducted the same in vivo LFP recordings in the OB of 3–5 month-old 3xTg mice and age-matched WT controls (Fig. 9a). Similarly to APP/PS1 mice, significantly increased power in the low-gamma band occurred in 3xTg mice (Fig. 9b-e). Moreover, microinjection of TGB significantly reduced gamma power in 3xTg mice and normalized the difference in gamma oscillations between 3xTg and WT mice (Fig. 9f). In addition to upregulated levels of the GABA_A_R α1 and β3 subunits (Fig. 9g-h, Additional file 1), which was also seen in APP/PS1 mice, 3xTg OB showed a significant increase in levels of GABA_A_R β2 and a decrease in GluR1 levels (Fig. 9g-h, Additional file 1). Furthermore, immunofluorescent staining confirmed the increased expression of the GABA_A_R α1 and β3 subunits (Fig. 9i-l). Taken together, we reason that enhanced gamma oscillations in the OB of AD models may function as a common electrophysiological marker of early AD.

## Discussion

**Fig. 10:**
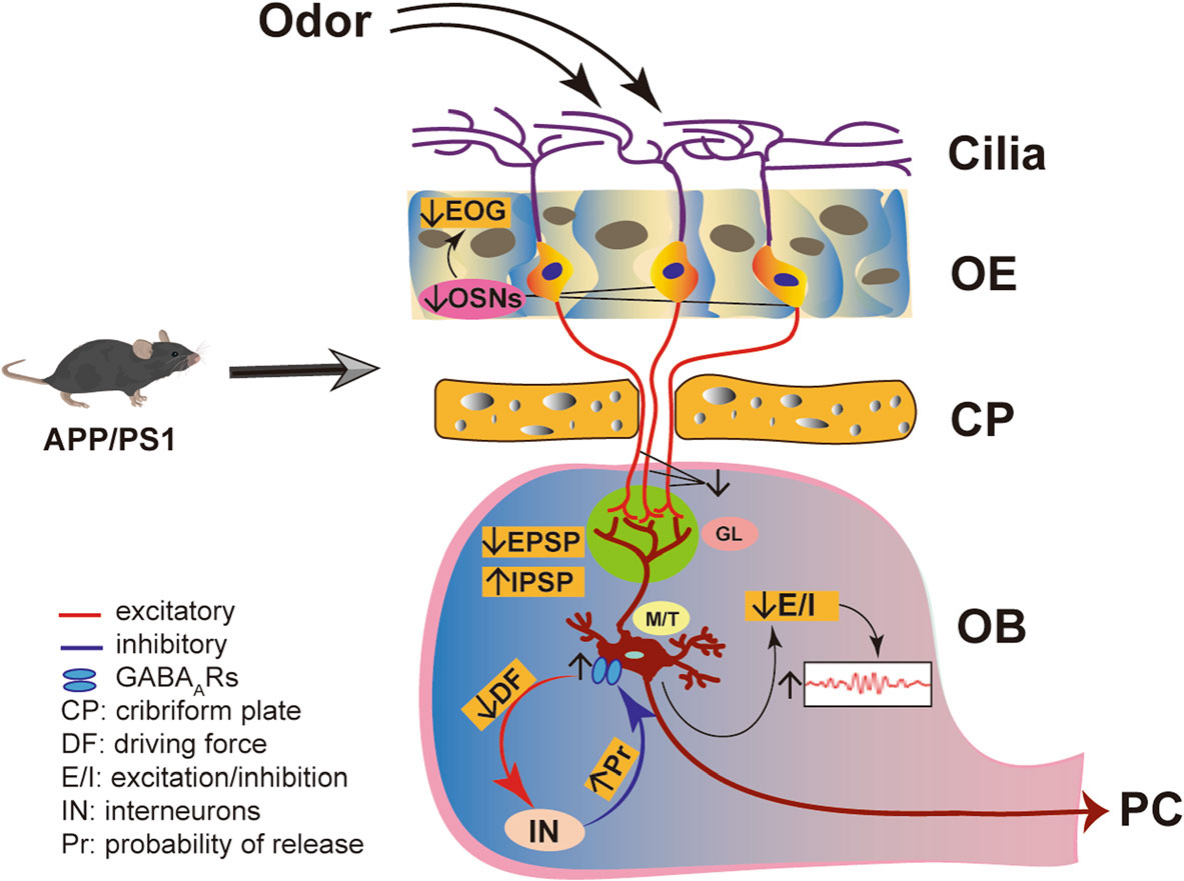
Schematic illustration of the neural mechanism underlying aberrant gamma oscillation. APP/PS1 mice exhibit a reduction in both the number of OSNs and in EOG amplitude (Fig. 5), resulting in a decreased OE → OB glutamatergic innervation (Fig. 3c, Fig. 4i-j) and subsequent reduction in the capacity of M/T cells to trigger GC and other interneurons to release GABA, accompanied with a compensatory increase in GABA_A_R subunits (Fig. 3d-i) that resulted in increased amplitude of inhibitory responses (Fig. 3b, Fig. 4f-g and n). Together, these alterations lead to reduction of E/I (Fig. 4o) and subsequent increase in gamma oscillation

The increasing awareness of olfactory dysfunction in people with prodromal neurodegenerative disease, including AD, and a deeper understanding of the physiology of olfactory perception [9, 91, 92], have made the olfactory neural network in rodents a useful model in which to investigate mechanisms and therapeutic targets of AD [23, 32, 93]. GABAergic dysfunction leading to excitatory and inhibitory imbalance has recently been proposed as a driver of AD pathogenesis [94], but the mechanisms underlying GABAergic dysfunction in AD are not yet fully characterized. Here we show that 3–5 month-old APP/PS1 and 3xTg mice present with altered olfactory behavior, associated with increased levels of GABA_A_R α1 and β3 subunits and gamma oscillations that can be ameliorated by TGB. We found that APP/PS1 mice exhibit altered OSN → M/T cell excitation and interneuron→M/T cell inhibition, resulting in disturbance of E/I balance and subsequent gamma oscillations in the OB (Fig. 10). Similar to how elimination of Aβ, the major pathological hallmark of AD, improves only certain symptoms of AD with limited pharmaceutical success thus far [95, 96], TGB can improve gamma oscillations and levels of GABA_A_R subunits did not ameliorate olfactory behavior in this study. Together, these results again suggest a necessity for multitarget-strategies for drug discovery in the treatment of AD.

Additionally, altered aPC-OB connectivity may also have negative impacts on the OB. Although both APP/PS1 and 3xTg mice exhibit an impairment of hippocampal-dependent spatial memory at 4 months [97, 98], oscillatory activity in the hippocampus remained unchanged (Fig. S6). Thus, the present study suggests that alterations in the gamma band of the OB, which are associated with abnormal olfactory behavior, may be useful as electrophysiological indicators of the early stages of AD. Interestingly, recent studies have shown that low-frequency light pulses at 40 Hz, i.e. in the gamma band, decrease levels of amyloid plaques [26], auditory and visual stimulation at low-gamma frequency (40 Hz) ameliorate AD pathology and improve cognition in an AD mouse model [30]. Therefore, altered gamma oscillations in the OB may have critical implications for the pathogenesis and treatment of AD.

Moreover, given that early-stage AD patients exhibit olfactory perceptual deficits before the manifestation of classical cognitive impairments, evaluation of olfaction might be used as a diagnostic indicator for potential AD. Considering that gamma oscillations can be detected in the human brain by magnetoencephalography (MEG) [99, 100], abnormal gamma wave in the OB of AD mice recommends a use of MEG to measure gamma oscillations in the early diagnosis of AD. Furthermore, neuronal oscillatory activity represents the neural basis of MRI [101]. Although currently MRI can only detect low frequencies, fast MRI and new MRI technologies currently available and also in development have the potential to map neural oscillations directly throughout the brain [102]. In addition, we found that altered levels of GABA_A_R expression co-occur with aberrant oscillations (Fig. 3 & Fig. 9), highlighting the potential of biochemical measurements to aid the diagnosis of early-stage AD.

Olfaction involves various stages of neural processing extending from sensory input at the OSNs of the nasal epithelium to the OB [17, 27]. In the present study, we observed a significant reduction in theta oscillations in APP/PS1 OB as well as in the number of mature OSNs and EOG amplitude, resulting in impaired excitatory innervation from the OE to OB and reduced excitatory response of M/T cells. These findings are in agreement with a notion that olfactory low-frequency theta activity reflects large synchronous volleys of afferent inputs from the nasal epithelium [52, 103]. We note that OSN axons mistarget in the OB of mice overexpressing human APP-Swedish mutation (hAPPsw) prior to the onset of plaques. Moreover, selective expression of hAPPsw in OSNs disturbs connectivity [104]. Therefore, the reduction in OSN number in the OE of 3–5 month-old AD mouse models might prove valuable not only for studying the pathological process of AD, but also for developing early diagnostic tools and for determining the efficacy of potential therapeutics against AD.

The interplay between inhibitory and excitatory neurons underlies the synchronous activities at fast gamma frequencies [70, 105]. Moreover, inhibitory interneurons involved in rhythm generation are segregated into distinct layers in the OB, and optogenetic silencing of the GC layer provides direct evidence of a causal role for GABAergic GCs in gamma oscillations in both anesthetized and awake animals [106]. Although the enhanced amplitude of IPSPs and mIPSCs and increased levels of GABA_A_R subunits suggest an increased postsynaptic inhibitory response of M/T cells, the reduction in PPR indicates a decreased presynaptic efficiency of GABAergic synaptic inhibition of the M/T cells in APPPS1 mice. The latter is also supported by wider synaptic clefts between GC- and M/T cell-dendrites in the EPL of 3–4 month-old APP/PS1 [32]. Together, these findings suggest a pre- and postsynaptic GABAergic disconnection in the OB of APP/PS1 mice. Furthermore, increasing GABA levels in the synaptic cleft by TGB treatment ameliorated aberrant gamma oscillations and levels of GABA_A_R subunit in APP/PS1 mice. We thus conclude that postsynaptic enhancement of inhibitory responses (mIPSC, eIPSC and field IPSP) together with possibly increased release probability indicated by a reduced PPR might represent compensation for a decrease in the capacity of M/T cells to trigger GC and other interneurons to release GABA (Fig. 10).

Likewise, TGB has been shown to reduce gamma oscillations in the human visual system [107], and a GABA_A_R agonist, muscimol, reduces gamma oscillations in OB slices of APP/PS1 mice [32, 107]. Moreover, a GABA_A_R antagonist, bicuculline, increases gamma oscillations [82]. We show in the present study that enhancing GABAergic inhibition by TGB reduces upregulated gamma oscillations and levels of GABA_A_R subunits in both APP/PS1 and 3xTg mice, elaborating the mechanisms underlying GABAergic defects that mediate aberrant gamma oscillations in AD model mice. We previously noted that reducing GABAergic inhibition rescued memory and olfactory deficits in aged 5xFAD mice, an AD model harboring five human familial AD mutations in the APP and PS1 genes [93]. This discrepancy may be because, in this earlier study, GABAergic inhibition was reduced by crossing Gad67 haploinsufficient mice with 5xFAD mice, generating an AD model with a congenital GABAergic defect that might cause alterations not commonly occurring in AD mice.

It is also worth noting that glutamate release from M/T dendrites can trigger recurrent excitation via the AMPARs and NMDARs, playing an important role in supporting oscillatory activity [27]. However, the levels of several subunits of these receptors remained unchanged in the OB of APP/PS1 mice (Fig. 3). On the other hand, an AMPAR subunit, GluR1, showed reduced levels in the OB of 3xTg mice, and this was accompanied by increased levels of GABA_A_R α1-, β2- and β3-subunit levels (Fig. 9). Therefore, although there are differences between APP/PS1 and 3xTg mice in the levels of certain excitatory and inhibitory receptors in the OB, increased gamma oscillations were observed in both AD mouse models. Given that the OB of young Tg2576 also demonstrates increased gamma oscillations [23], an increase in gamma oscillations in the OB could be an early biomarker prior to the onset of AD.

Functional connectivity between distant brain structures is fundamental in coordinating neuronal informational communication during sensory processing. It has been shown that proper fast neuronal synchronization between the PC and OB is critical for olfactory behavior [27, 86, 108]. Utilizing CSC and PSC analysis, we found significantly decreased synchronization between simultaneously recorded LFPs in the OB and aPC of APP/PS1 mice in both the beta- and gamma-frequency ranges. The beta frequencies synchronize over long conduction delays for higher-level interactions between distant structures, whereas gamma frequencies involve relatively local computations [109, 110]. Though the functional role of beta and gamma oscillations in the olfactory cortex is not yet well understood, we uncovered, in the present study, reduced frequency-domain consistency or phase synchronization in both the beta and gamma bands between the OB and aPC. These results prove an impairment of large-scale and relatively dampened local-scale network activity in the olfactory pathway of 3–5 month-old APP/PS1 mice. PAC between brain structures is an innovative evaluation of neural coding schemes playing a critical role in the support and promotion of network activity [49, 111]. The significantly reduced theta–gamma PAC between the OB and aPC in APP/PS1 mice suggests an impairment in the neural interactions governing information gating and communication between the OB and aPC.

Although anesthetic agents might have a profound effect on E/I balance and network state [112, 113], previous studies have shown that the OB oscillatory activities elicited by odor stimulation were comparable in urethane-anesthetized and awake rodents [87]. Similarly, a recent study reported that the PC responses to odor were largely unchanged by anesthesia [114]. Thus, abnormal oscillatory power and connectivity in anesthetized mice can be used to evaluate olfactory deficits in early AD pathology. In addition, c-Fos staining result suggests that the aPC of APP/PS1 mice is less activated under physiological conditions without specific stimulation (Fig. S7). Together with reduced M/T cell excitation, this might indicate a possible reduction in M/T → aPC activation and subsequent aPC → OB feedback projections that would contribute, at least in part, to abnormal connectivity between the OB and aPC in APP/PS1 mice.

Finally, a γ-secretase inhibitor, LY-411575, also ameliorated the abnormality in gamma oscillations in 3–5 month-old APP/PS1 mice, suggesting that toxicity of Aβ oligomers contributes to the impairment of olfactory network function. Similarly, several studies have shown that Aβ impairs gamma oscillations. For example, previous studies showed that Aβ1–42 can induce dysfunction in gamma synchronization [115, 116], and increases Aβ deposition in the OB of APP/PS1 mice, which can impair gamma-oscillations [32]. However, given that clinical trials aimed at reducing Aβ production and/or increasing Aβ clearance have failed thus far [117–120], it is tempting to speculate that components of the GABAergic signaling pathway may prove to be better potential targets for the treatment of early olfactory network abnormalities in AD [121–123].

## Conclusions

Our findings provide novel insight into the impairment of gamma oscillations observed in the OB of AD mouse models. The alterations in gamma activities and levels of GABA-signaling related proteins are apparent in 3–5 month-old AD mice, which may represent early signs of AD pathogenesis. Furthermore, amelioration of gamma oscillations by an FDA-approved anticonvulsant, TGB, suggests that this medicine might be exploited to counteract damage of olfactory dysfunction caused by impaired GABAergic activity. Thus, the present study may offer a new window for early diagnosis of AD and provide a novel therapeutic possibility against AD pathogenesis.

## Supplementary Information

**Additional file 1: **Original Western blotting results.

## Data Availability

All data generated or analyzed during this study are included in this article and its supplementary information files.

## Abbreviations

aCSF: Artificial cerebral spinal fluid
AD: Alzheimer’s disease
ANOVA: Analysis of variance
aPC: Anterior piriform cortex
APV: Amino-phosphonovaleric acid
APP: Amyloid precursor protein
Aβ: Amyloid-beta
AMPA: α-amino-3-hydroxy-5-methyl-4-isoxazolepropionic acid
CTF: APP C-terminal fragments
CNQX: Cyanquixaline
CSC: Cross-spectral coherence
EOG: Electro-olfactogram
EPL: External plexiform layer
GAT1: GABA transporter 1
GABA: Gamma-aminobutyric acid
GBZ: GABAzine
GL: Glomerular layer
GCs: Granule cells
hAPPsw: Human amyloid precursor protein Swedish mutations
INs: Interneurons
IP: Intraperitoneal
LFP: Local field potential
M/T: Mitral/tufted
MCL: Mitral cell layer
mEPSC: Miniature excitatory postsynaptic current
mIPSC: Miniature excitatory postsynaptic current
NMDA: N-Methyl-d-aspartic acid
OB: Olfactory bulb
OE: Olfactory epithelium
OFT: Open field test
OMP: Olfactory marker protein
OSNs: Olfactory sensory neurons
PAC: Phase-amplitude coupling
PBS: Phosphate-buffered saline
PC: Piriform cortex
PCR: Polymerase chain reaction
PFA: Paraformaldehyde
PPR: Paired-pulse ratio
PS1: Presenilin 1
PSC: Phase synchronization clustering
PV: Parvalbumin
RT: Room temperature
SDS-PAGE: Sodium dodecyl sulphate-polyacrylamide gel electrophoresis
SWSh: Spatial win-shift
TEA-Cl: Tetraethylammonium chloride
TGB: Tiagabine
TTX: Tetrodotoxin
WT: Wild-type
