# Supplementary material for: Enhancing GABAergic signaling ameliorates aberrant gamma oscillations of olfactory bulb in AD mouse models
Source: Mol Neurodegener. 2021 Mar 4;16:14. doi: 10.1186/s13024-021-00434-7 (PMC7934466; doi:10.1186/s13024-021-00434-7)

Fig. 3d

**mGluR5**


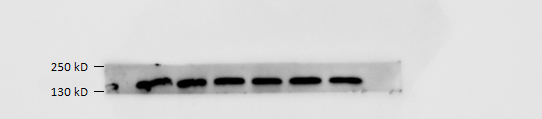


**GluR1**


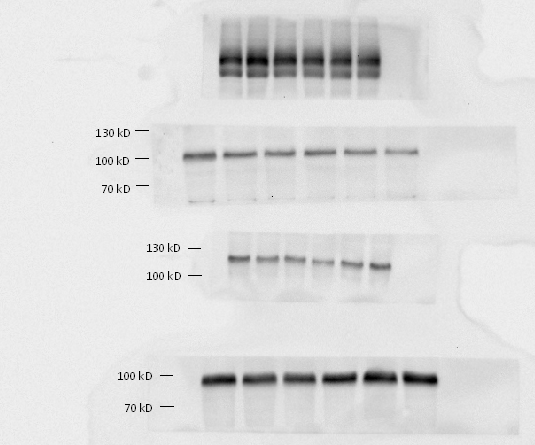


**NMDAR**

**GluR2**

**GABA_B1_R**


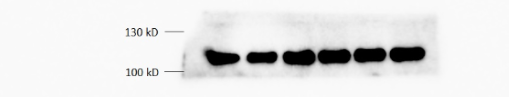


**GABA_B2_R**


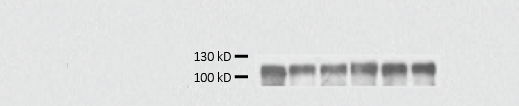


**GABA_A_R β2**


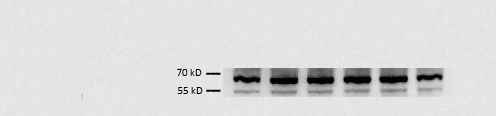


**GABA_A_R β3**


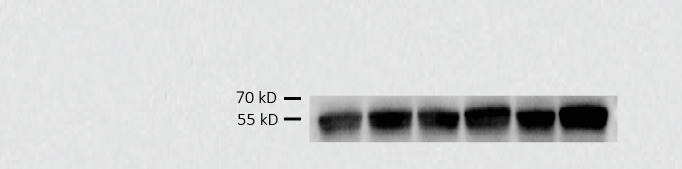


**GABA_A_R α5**


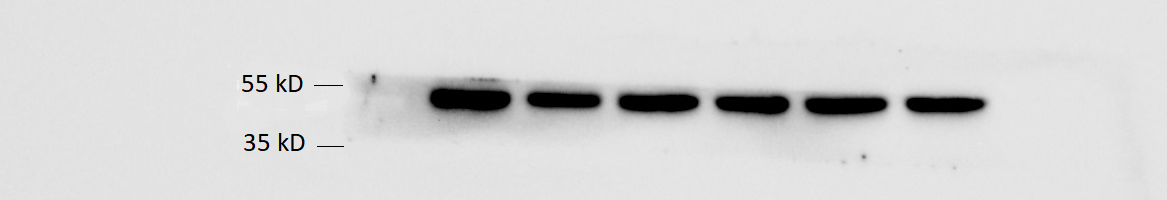


**GABA_A_R α1**


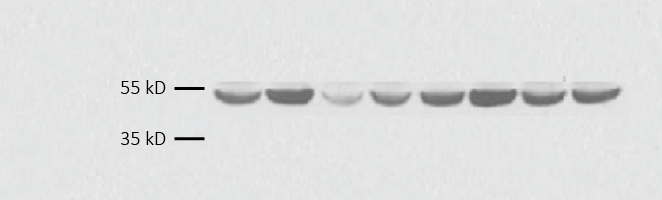


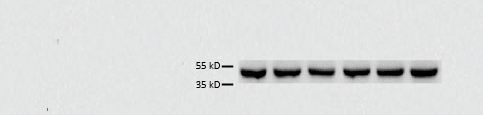


**γ-Tubulin**

Fig. 4d

**GAT3**


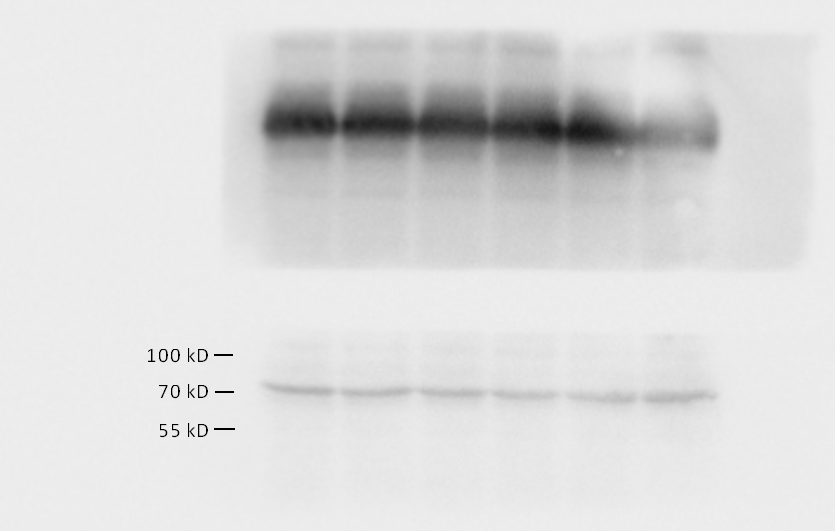


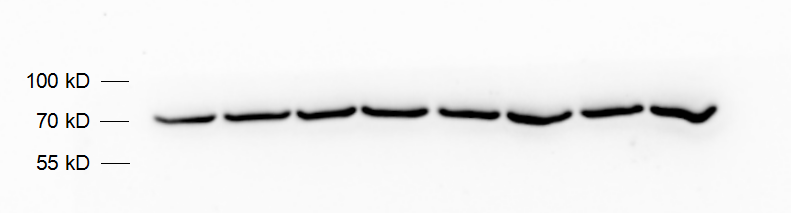


**GAT1**

**GAD65/67**


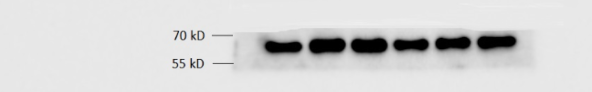


**VGAT**


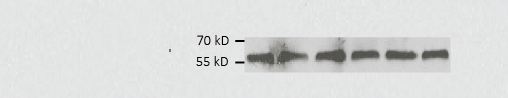


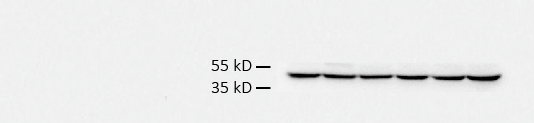


**γ-Tubulin**

Fig. 6f


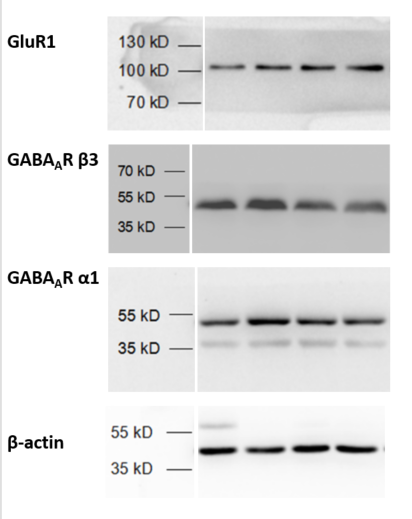


Fig. 9g


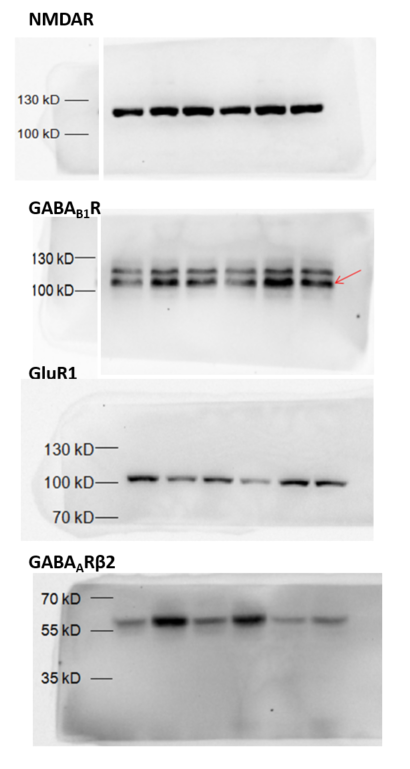


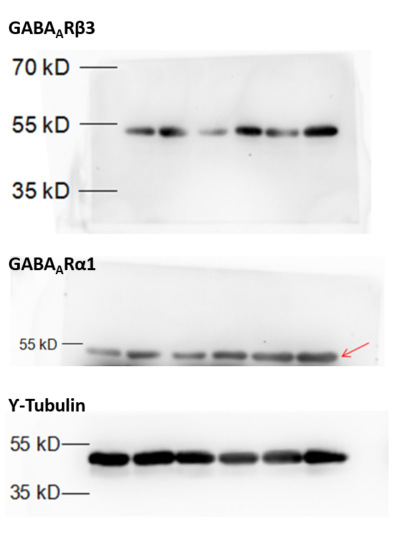

Supplement: Supplementary file 1 — Additional file 1: Original Western blotting results. [file 13024_2021_434_MOESM1_ESM.doc]
